# Design, Synthesis, and Antimalarial Evaluation of New Spiroacridine Derivatives

**DOI:** 10.3390/antibiotics14121214

**Published:** 2025-12-02

**Authors:** Misael de Azevedo Teotônio Cavalcanti, Sonaly Lima Albino, Karla Joane da Silva Menezes, Wallyson Junio Santos de Araújo, Fernanda de França Genuíno Ramos Campos, Malu Maria Lucas dos Reis, Inês Morais, Denise Maria Figueiredo Araújo Duarte, Igor José dos Santos Nascimento, Valnês da Silva Rodrigues-Junior, Fátima Nogueira, Ricardo Olímpio de Moura

**Affiliations:** 1 Programa de Pós-Graduação em Ciências Farmacêuticas (PPGCF), Universidade Estadual da Paraíba (UEPB), Campina Grande 58429-500, PB, Brazil; misaelazevedo.2015@gmail.com (M.d.A.T.C.); sonaly.albino@hotmail.com (S.L.A.); igorjsn@hotmail.com (I.J.d.S.N.); 2 Laboratório de Desenvolvimento e Síntese de Fármacos (LDSF), Universidade Estadual da Paraíba (UEPB), Campina Grande 58429-500, PB, Brazil; menezeskarla5@gmail.com (K.J.d.S.M.); wallyson.araujo@aluno.uepb.edu.br (W.J.S.d.A.); fernanda.campos@aluno.uepb.edu.br (F.d.F.G.R.C.); malureisduarte@gmail.com (M.M.L.d.R.); 3 Departamento de Química, Universidade Estadual da Paraíba (UEPB), Campina Grande 58429-500, PB, Brazil; 4 Global Health and Tropical Medicine (GHTM), Associate Laboratory in Translation and Innovation Towards Global Health, LA-REAL, Instituto de Higiene e Medicina Tropical (IHMT), Universidade NOVA de Lisboa (UNL), Rua da Junqueira 100, 1349-008 Lisboa, Portugal; ines.morais@ihmt.unl.pt (I.M.); dduarte@ihmt.unl.pt (D.M.F.A.D.); 5 Programa de Pós-Graduação em Produtos Naturais e Sintéticos Bioativos (PgPNSB), Departamento de Ciências Farmacêuticas, Universidade Federal da Paraíba (UFPB), João Pessoa 58051-900, PB, Brazil; valnesjunior@cbiotec.ufpb.br

**Keywords:** acridine, malaria, tropical parasitic diseases, synthesis, molecular docking, molecular dynamics, MM-PBSA, dihydrofolate reductase, thymidylate synthase

## Abstract

**Background/Objectives:** Malaria is a tropical disease mainly caused by *Plasmodium falciparum* and represents a global public health problem, with over 200 million cases and 500 thousand deaths reported worldwide. Considering its treatment limitations, it is essential to develop new compounds against malaria. In this context, acridine derivatives are privileged structures. **Methods:** Thus, new spiroacridines containing *N*-acylhydrazone (**AMTAC**) and *N*-phenylacetamide (**ACMD**) were synthesized and evaluated in malaria and cytotoxicity assays, as well as *in silico* studies. **Results:** As a result, five spiroacridines showed inhibitory activity over 70% against the *P. falciparum* 3D7-GFP strain at 10 μM, along with an IC_50_ range of 2–4 μM. After a brief Structure–Activity Relationship (SAR) analysis, it was observed that the spiroacridine structure must be associated with the hydrazone moiety to successfully inhibit parasite growth. In addition, these molecules presented promising resistance profile, with selectivity for the parasite. After computational studies, spiroacridines showed better affinity with dihydrofolate reductase (DHFR), overcoming the quadruple mutant resistance to pyrimethamine, with more stability in complex with the enzyme. **Conclusions:** Therefore, the potential of spiroacridines against malaria, with moderate resistance and selectivity profile, as well as DHFR inhibition greater than pyrimethamine, was confirmed.

## 1. Introduction

Malaria is a tropical disease transmitted through the bite of female Anopheles mosquitoes and caused by protozoa of the *Plasmodium* genus, of which five species can infect humans (*P. falciparum, P. vivax, P. malariae, P. ovale*, and *P. knowlesi*) [1]. Among these, *P. falciparum* is responsible for 97% of cases reported globally and is notorious for inducing severe clinical conditions due to its propensity to “sequester” infected erythrocytes in the microvasculature of vital organs, which can lead to cerebral malaria and multiple organ dysfunction [2,3].

Thus, malaria remains a public health issue of high global impact. According to recent data published by the World Health Organization, approximately 263 million cases and 597,000 deaths from malaria were reported worldwide in 2023, of which approximately 95% of these fatalities occurred in the African Region, mainly in four countries of Sub-Saharan Africa: Nigeria, the Democratic Republic of the Congo, Niger, and the United Republic of Tanzania, which account for more than half of global malaria-related deaths [4].

The chemotherapeutic treatment used to control malaria comprises an arsenal of drugs categorized according to the specific stage of the parasite’s life cycle in which they act. Thus, they consist of gametocides (artemisinin, chloroquine), blood schizonticides (halofantrine, sulfadoxine, mefloquine, and quinine), tissue schizonticides (pyrimethamine and primaquine), and sporonticides (pyrimethamine and primaquine) [5]. Nevertheless, several limitations are associated with the drugs available for the treatment of malaria, such as low efficacy, high toxicity, and the growing prevalence of resistant parasites, a consequence directly related to the rising mutation rate of the *Plasmodium* genome. As a result, there has been a continuous increase in the rates of global malaria cases since 2015 [6,7]. The spread of *Plasmodium* resistance to the current chemotherapeutic arsenal underscores the urgent need for new antimalarial agents with novel mechanisms of action. Therefore, there is an urgent demand for the development of novel chemical compounds that can perform safe and effective antimalarial activity against drug-sensitive and drug-resistant *Plasmodium*, targeting dual and multistage phases of the parasite life cycle [8].

Given this premise, the privileged acridine nucleus stands out. It is the central core and the main pharmacophoric group of quinacrine, the first synthetic antimalarial introduced into clinical practice, and currently employed as a scaffold for the design and synthesis of novel promising antimalarial acridine derivatives or analogs. The acridine nucleus demonstrates remarkable antimalarial potential, performing this activity through multiple mechanisms of action, such as interaction with DNA, inhibition of parasitic topoisomerase II, disruption of the mitochondrial bc1 complex, and interference with hemozoin formation [9,10,11].

In this context, acridine, specifically spiroacridine compounds, has attracted the interest of researchers due to its mechanisms of action, positioning it as a potential chemotherapeutic agents [12]. Thus, spiroacridine derivatives have been investigated by our research group and stand out due to their antitumor [13,14,15,16,17,18,19], antileishmanial [20,21,22], and antifungal [23] activities.

Based on this premise, this work proposes the synthesis and evaluation of the antimalarial activity and mechanism of action of spiroacridines. This study focused on the rational design of new spiroacridine derivatives, their synthesis and structural characterization, and the investigation of their antimalarial activity and parasite selectivity of the obtained compounds, compared with their respective *N*-acylhydrazone reaction intermediates. For this purpose, *in vitro* assays were carried out against different strains of *P. falciparum* that are sensitive and resistant to commercial drugs, along with *in silico* theoretical studies of mechanism of action and pharmacokinetic prediction.

## 2. Results

### 2.1. Design of Compounds

**Figure 1:**
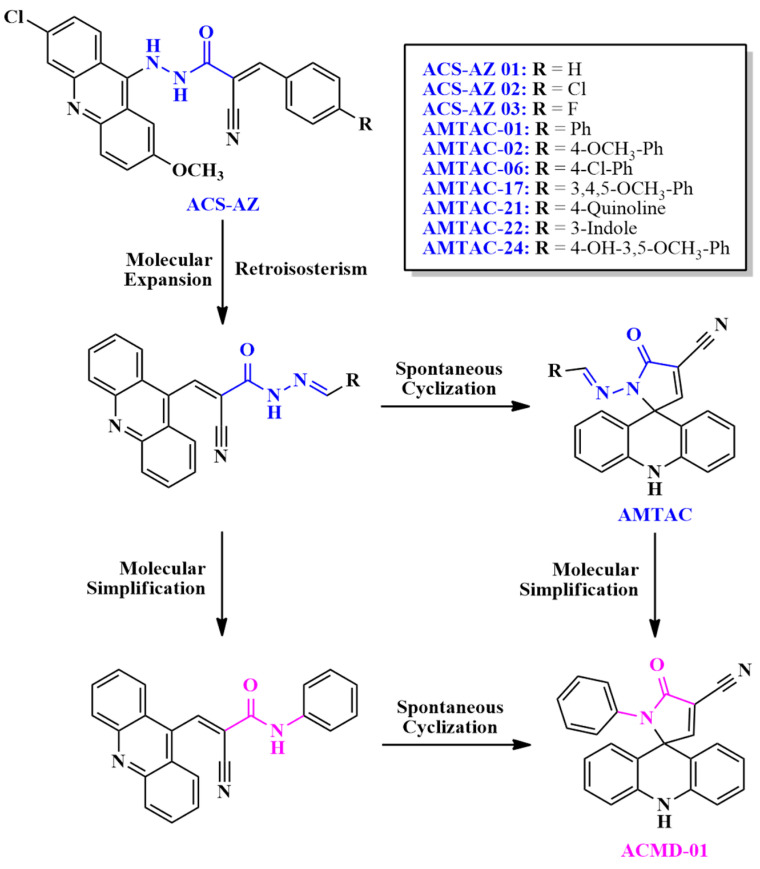
Design of *N*-acylhydrazone and acetamide spiroacridines. The acetohydrazide and *N*-acylhydrazone moieties are represented in blue, whereas acetamide is represented in pink, as well as the compound’s names which contains each group are represented in the corresponding color.

Considering the significant antimalarial activity and selectivity toward *P*. *falciparum* W2 of the hydrazine compounds **ACS-AZ** series (Figure 1), which showed IC_50_ of 0.90–3.20 μM compared to primaquine (IC_50_ = 1.70 μM) [24], their structures were used to design new acridine derivatives (Figure 1). For this, classic tools of medicinal chemistry were used to perform chemical modifications. In this way, molecular expansion and retroisosterism were applied to obtain the *N*-acylhydrazone spiroacridines (**AMTAC**), by reverting the position of acetohydrazide (**ACS-AZ 01-03**), highlighted in blue, and expanding this moiety with the addition of an sp^2^ carbon. This modification was performed to assess the relevance of acetohydrazide moiety and to increase the chain, with the conversion of hydrazine to hydrazone, besides the direct attachment of the amino group at the 9-position of the acridine ring present in compounds **ACS-AZ 01-03**. After the retroisosterism modification, the acetohydrazide group became more labile, which can possibly justify the spontaneous cyclization generating the spiroacridine ring [12]. Subsequently, using molecular simplification and bioisosterism, the *N*-phenylacetamide spiroacridine (**ACMD-01**) was designed by removal of the imine group after the conversion of acetohydrazide to acetamide, allowing the assessment of the relevance of the hydrazone moiety.

### 2.2. Synthesis and Structural Elucidation

**Figure 2:**
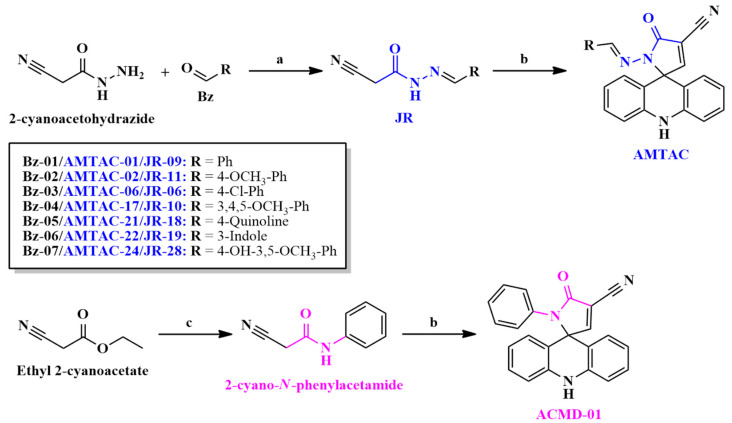
Synthesis of *N*-acylhydrazone and acetamide compounds. Reaction conditions are as follows: (a) EtOH, AcOH, r.t.; (b) 9-Acridinecarboxaldehyde, EtOH, Et_3_N, 78 °C; and (c) DMF, aniline, 160 °C. The acetohydrazide and *N*-acylhydrazone moieties are represented in blue, whereas acetamide is represented in pink, as well as the compound’s names which contains each group are represented in the corresponding color.

For the synthesis of **AMTAC** compounds, intermediates **JR** (Figure 2) were obtained by aldol condensation of 2-cyanoacetohydrazide and different benzaldehydes in ethanol under acidic conditions at room temperature [25,26]. Then, these compounds were submitted to a Knoevenagel condensation with 9-acridinecarboxaldehyde (Figure 2). **AMTAC-01**, **AMTAC-02**, **AMTAC-06,** and **AMTAC-17** were already synthesized in previous works of the research group, in a basic environment using ethanol as the solvent under reflux [12,16,19]. The same protocol was performed for the new spiroacridines **AMTAC-21**, **AMTAC-22,** and **AMTAC-24**, resulting in yields of 69–90%. As for the synthesis of spiroacridine **ACMD-01**, 2-cyano-*N*-phenylacetamide was acquired by an amidation reaction of ethyl 2-cyanoacetate with aniline and submitted to a Knoevenagel condensation with 9-acridinecarboxaldehyde (Figure 2), following the same protocol of Albino et al. (2025) [22].

Among these compounds, some were synthesized in previous works, and their structural elucidation was already carried out, such as **JR-09** [26], **JR-18** [27], **JR-19** [25], as well as **AMTAC-01**, **AMTAC-02**, **AMTAC-06**, **AMTAC-17,** and **ACMD-01** [12,16,19,22]. However, the other compounds are reported for the first time, and their structures were confirmed through ^1^H-NMR, ^13^C-NMR, and IR spectroscopy, as well as mass spectrometry, except for the intermediates **JR-11**, **JR-06**, **JR-10**, and **JR-28**. These data were consistent with the expected chemical structures, as described in the Supplementary Material (Figures S1–S22).

In all ^1^H-NMR spectra of **JR**, two characteristic singlets were observed, one in the range of 11.8–12.0 ppm corresponding to NH group, and another at 3.7–4.3 ppm attributed to methylene hydrogens, besides the aromatic shifts at 6.97–8.96 ppm. The duplicate peaks can be justified by the possible formation of anti- and synperiplanar conformers, common in *N*-acylhydrazones [26,28,29]. As for ^1^H- and ^13^C-NMR spectra of **AMTAC-21**, **AMTAC-22,** and **AMTAC-24**, characteristic hydrogen peaks of the spiroacridine scaffold, such as NH (9.85–11.49 ppm), pyrrole alkene (8.57–8.74 ppm), and imine (8.32–8.59 ppm), as well as carbon shifts in carbonyl (160–161 ppm), imine (150–160 ppm), alkene (148 ppm), and the quaternary carbon (68–69 ppm) were identified. Moreover, both **AMTAC** and **JR** series of compounds were represented in the *E* isomer of the imine bond since this configuration is predominant in *N*-acylhydrazones of aromatic aldehydes, whereas the Z isomer is less stable and short-lived due to steric hindrance [29].

Along with NMR, the infrared spectroscopy results were as expected, with identification of axial deformations of secondary amide (NH), aromatic C-H (sp^2^), amide carbonyl (C=O), and aromatic C=C, as well as bending vibrations of amide (N-H) and aromatic rings (C-H) with *ortho*, *meta,* or *para* substitutions. Finally, mass spectrometry corroborated the molecular weight and chemical formula of the synthesized compounds since the calculated mass values were similar to the ion molecular peaks, with a slight difference between the experimental and calculated *m/z* ratios in positive mode. In summary, these characterization data are in agreement with previous studies performed with these kind of compounds [12,16,18,19,22].

### 2.3. Antimalarial Activity Against Asexual Blood Stages

In order to assess the antiplasmodial activity of the compounds, a screening with fixed concentrations at 10 μM was performed against unsynchronized cultures of the asexual blood stages of *P*. *falciparum* 3D7HT-GFP (hereafter referred to as 3D7-GFP). The results obtained with this assay were represented as a percentage of inhibition of *P*. *falciparum* 3D7-GFP culture, as displayed in Table 1. The **JR intermediates** were also evaluated in this assay in order to assess the relevance of the spiroacridine scaffold.

For this experiment, compounds with inhibition percentages over 70% at the maximal concentration (10 μM) were associated with considerable antiplasmodial activity. In this way, almost all the spiroacridines, except **AMTAC-06**, **AMTAC-24,** and **ACMD-01**, showed antimalarial potential, with percentages over 90%. However, the *N*-acylhydrazone intermediates (**JRs**) and the spiroacridine acetamide **ACMD-01** were inactive against malaria due to inhibition results under 20%.

Afterward, the five compounds **AMTAC-01**, **AMTAC-02**, **AMTAC-17**, **AMTAC-21,** and **AMTAC-22**, which showed inhibition percentages over 70%, at 10 μM, were submitted to the determination of Half-Maximal Inhibitory Concentration (IC_50_) against the following three *P*. *falciparum* strains: 3D7-GFP (chloroquine-sensitive), Dd2 (chloroquine-resistant), and MRA-1240 (artemisinin-resistant) (Table 1). For validation of these assays, chloroquine was used as a positive control, and the IC_50_ values were comparable to those of other studies [30,31]. The concentration–response curves are displayed in Figures S23–S25 (Supplementary Material).

All compounds showed IC_50_ values around 2.0–4.0 μM against the three *Plasmodium falciparum* strains evaluated (Table 1). Although these results are less potent compared to chloroquine, which showed an IC_50_ of 0.02–0.26 μM [30,31], the resistance index (RI = IC_50_ of resistant strain/IC_50_ of susceptible strain) of spiroacridines showed favorable values, between 0.6 and 1.3, compared to 11.0 and 7.9 for the standard drug. Since lower RI values are associated with less resistance of the parasite strain against the compounds assessed, spiroacridines showed the ability to overcome resistance towards chloroquine.

### 2.4. Cytotoxicity Against Monkey Kidney Cells (Vero E6)

**Figure 3:**
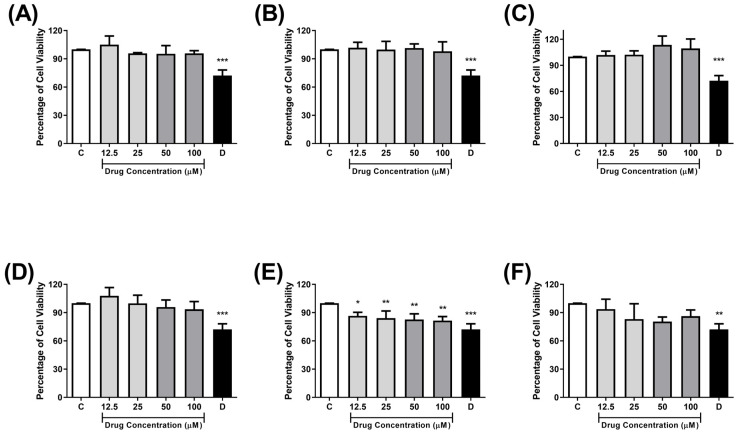
Cytotoxic effects of test compounds **AMTAC-01** (**A**), **AMTAC-02** (**B**), **AMTAC-17** (**C**), **AMTAC-21** (**D**), **AMTAC-22** (**E**), and **chloroquine** (**F**) on Vero E6 cells. C, Control: 1% DMSO-treated wells, considered as 100% cell viability. D: 10% DMSO. Results were expressed as mean of cell viability ± standard deviation of four independent experiments performed in triplicates. *** *p* < 0.001, ** *p <* 0.01, and * *p <* 0.05, compared to the corresponding control group. Data were evaluated by ANOVA, followed by Tukey’s post-test using GraphPad Prism 8.0.

In the following stages, to assess the selectivity of compounds against *P*. *falciparum* over mammalian cells, the cytotoxicity against monkey kidney cells (Vero E6) was determined through MTT assay after 72h of treatment. Also, the selectivity index (SI) of compounds was calculated by the ratio between a compound’s cytotoxicity against Vero E6 cells and its antiplasmodial activity (SI = Vero E6 IC_50_/*P*. *falciparum* IC_50_). All IC_50_ values and selectivity index (SI) are represented in Table 2. Despite **AMTAC-22** presenting some toxicity, with a 15–20% reduction in cell viability at all concentrations evaluated (12.5–100 μM), all spiroacridines showed safety towards the kidney cells, as well as the standard drug chloroquine (Figure 3), with SI values over 20.

### 2.5. Proposing the Mechanism of Action Through Molecular Docking

To suggest the possible mechanism of action involved in the antimalarial activity determined *in vitro*, the most potent compounds (**AMTAC-01**, **AMTAC-02**, **AMTAC-17**, **AMTAC-21,** and **AMTAC-22**) were selected for molecular docking. For this purpose, pharmacological targets essential for parasite survival were chosen for analysis, including dihydroorotate dehydrogenase (DHODH), bifunctional dihydrofolate reductase-thymidylate synthase (DHFR), purine nucleoside phosphorylase (PNPase), topoisomerase II (Topo II), prolyl-tRNA synthetase (ProRS), lactate dehydrogenase (LDH), enoyl acyl carrier protein reductase (ENR), falcipain-2 (FP2), and falcipain-3 (FP3) of *P*. *falciparum*.

To validate the methodology, the Root Mean Square Deviation (RMSD) values for DHODH, DHFR, PNPase, ProRS, LDH, ENR, FP2, and FP3 were in the range of 0.41–1.08 Å, below the 2.0 Å cutoff. As for Topo II, in the absence of a co-crystallized ligand, the system was validated through the interaction profile of the inhibitor with the catalytic residues within the active site, as described in the literature [32]. The binding affinity of each compound was assessed using the Fitness Score, with the results summarized in Table 3. Among the eight molecular targets evaluated, the most likely involved in the antimalarial activity of the spiroacridines is DHFR, which presented higher score values compared to the co-crystallized standard drug pyrimethamine.

**Figure 4:**
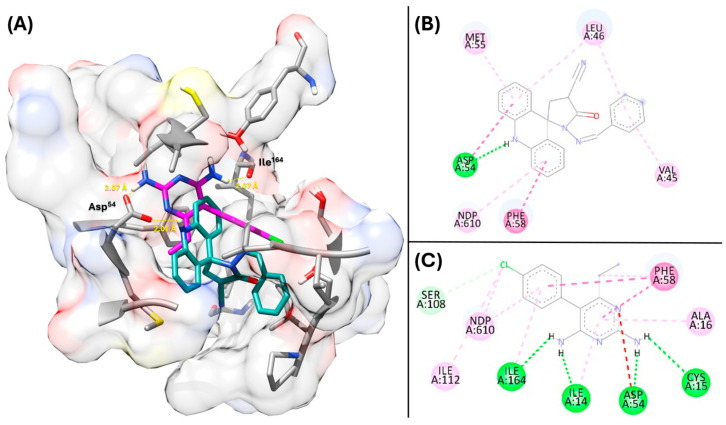
**Three-dimensional** representation of DHFR complex with pyrimethamine (magenta) and **AMTAC-01** (cyan) (**A**). Two-dimensional interaction diagram of DHFR complexes with **AMTAC-01** (**B**) and pyrimethamine (**C**). Legend: dark green = hydrogen bond; light green = carbon–hydrogen interaction; dark pink = π-π stacking; light pink = π-alkyl and alkyl interactions; and red = unfavorable acceptor–acceptor; All carbon, nitrogen, oxygen and sulfur atoms are represented in gray, blue, red and yellow color, respectively.

All spiroacridines showed greater affinity with both DHFR enzymes, wild-type (*wt*DHFR) and quadrupole-mutated (*qm*DHFR), compared to pyrimethamine, with higher FitScore values, except for **AMTAC-02** with the mutated enzyme. The binding mode showed that hydrophobic interactions are essential for affinity with the active site, including π-π stacking and alkyl–aryl interactions. However, the H-bond with some essential residues, such as Asp^54^ and Ile^164^, can improve complex stabilization [33,34], considering the high score of **AMTAC-01** (70.45) (Figure 4). Although **AMTAC-21** and **AMTAC-22** showed higher scores, **AMTAC-01** was more active in the *in vitro* assays due to its higher potency and lower toxicity. Thus, this compound was selected for further *in silico* analysis through MD simulations and MM-PBSA calculations.

### 2.6. Molecular Dynamics (MDs) Simulations to Propose the Target and Insights to Overcome Resistance

**Figure 5:**
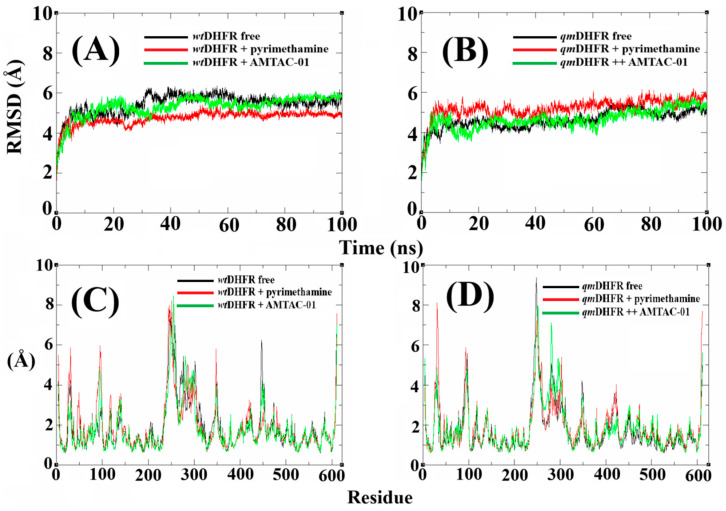
Plots of MD simulation in a 100 ns trajectory: RMSD of *wt*DHFR (**A**) and *qm*DHFR (**B**), and RMSF of *wt*DHFR (**C**) and *qm*DHFR (**D**), highlighting the free proteins (black lines) and in complex with the standard compound (red line) and **AMTAC-01** (green line).

After molecular docking, the enzymes *wt*DHFR and *qm*DHFR, which provided the best results, were chosen for MD simulations to suggest their potential as drug targets for this chemical class of compounds. In addition, the MD simulations were performed with the complexes with **AMTAC-01**, as it is expected to be the most active compound in the work. Then, after 100 ns of simulation, the compound **AMTAC-01** showed great stability in the RMSD plots at the binding site of both enzymes (Figure 5A,B). For *wt*DHFR, the RMSD value for the free enzyme was around 6 Å, stabilizing after 40 ns of simulation. In addition, the complexes with pyrimethamine and **AMTAC-01** showed similar variations, ranging from 4 to 5 Å (Figure 5A). On the other hand, for *qm*DHFR, the free protein showed RMSD around 5 Å, and the complexes with pyrimethamine and **AMTAC-01** were around 4 Å (Figure 5B). Also, it is clear that the lowest stability of pyrimethamine at *qm*DHFR compared to *wt*DHFR, and the improved stability of **AMTAC-01,** may suggest DHFR as the main drug target, as well as provide additional information that this spiroacridine can overcome resistance to pyrimethamine.

Next, for the Root Mean Square Fluctuation (RMSF) results (Figure 5C,D), a similar standard is observed in the simulations of the free DHFR and in the complexes with pyrimethamine and **AMTAC-01**. The fluctuations of the residues were up to 10 Å, with higher intensity in the region of the binding site between residues 50–150 for the complexes with *wt*DHFR, highlighting the permanence of the compounds at the binding site (Figure 5C). Nevertheless, for the complexes with *qm*DHFR (Figure 5D), a minor fluctuation in the region of the binding site for the complex with pyrimethamine is expected due to resistance. Furthermore, the permanence of **AMTAC-01** at the binding site is clear, suggesting once again that the compound can overcome resistance to pyrimethamine.

**Figure 6:**
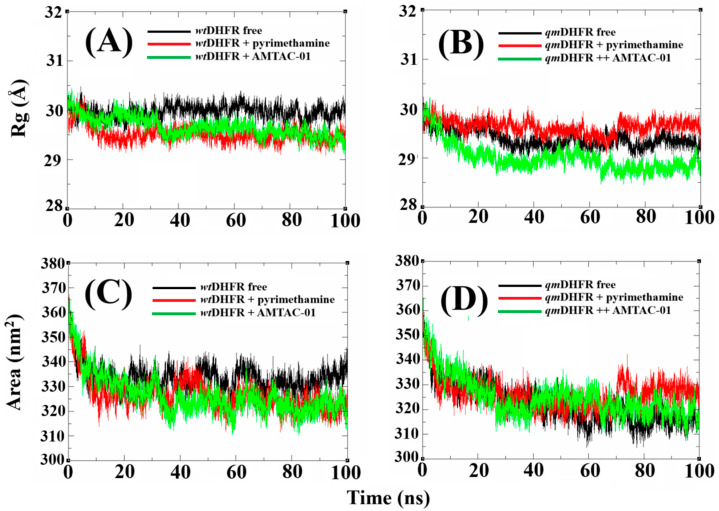
Plots of MD simulation in a 100 ns trajectory: R_g_ of *wt*DHFR (**A**) and *qm*DHFR (**B**), and SASA of *wt*DHFR (**C**) and *qm*DHFR (**D**), highlighting the free proteins (black lines) and in complex with the standard compound (red line) and **AMTAC-01** (green line).

The plot of the radius of gyration (R_g_) (Figure 6A,B) suggests the rigidity and compactness of the protein. In this way, for *wt*DHFR (Figure 6A), the complexes with pyrimethamine and **AMTAC-01** presented similar values throughout the simulations, around 30 Å, stabilizing around 40 Å. However, for *qm*DHFR (Figure 6B), it is clear that **AMTAC-01** allowed better stability compared to pyrimethamine, around 29 and 31 Å, respectively. Furthermore, the Solvent Accessible Surface Area (SASA) plots (Figure 6C,D) provided information about the influence of water molecules on the stability of the complexes. In fact, both compounds in complex with *wt*DHFR (Figure 6C) showed great stability relative to the free protein, with values around 330 nm^2^, stabilizing after 20 ns of simulation. Similar to previous results, for the complexes with *qm*DHFR (Figure 6D), after 60 ns of simulation, trajectory deviations occur for the complex with pyrimethamine, with values around 340 nm^2^, while the complex with **AMTAC-01** maintains stability similar to the free protein, with values around 320 nm^2^. Once again, our findings prove that DHFR is the main target, and the information about the resistance of DHFR against pyrimethamine suggests that **AMTAC-01** can overcome this mechanism of resistance.

**Figure 7:**
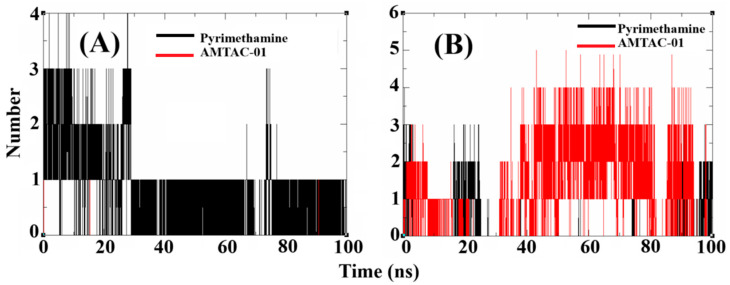
Plots of MD simulation in a 100 ns trajectory: H-bond around the trajectory for *wt*DHFR (**A**) and *qm*DHFR (**B**), highlighting the standard compound (black line) and **AMTAC-01** (red line).

Finally, the analysis of the H-bond plots (Figure 7A,B) for the compounds in complex with *wt*DHFR and *qm*DHFR highlighted up to four H-bond interactions at the binding site for pyrimethamine in complex with *wt*DHFR and up to two for **AMTAC-01** (Figure 7A). However, for the complexes with *qm*DHFR (Figure 7B), a decrease in the H-bond interactions was observed for pyrimethamine, showing up to three interatctions and a progressive decrease throughout the trajectory. In contrast, for the complex with **AMTAC-01**, the results showed an increase in H-bond interactions throughout the trajectory, with a maximum of five interactions. These data confirm the resistance of *qm*DHFR to pyrimethamine and once again suggest the importance of the molecular scaffold of spiroacridine **AMTAC-01** in overcoming resistance against this target.

### 2.7. Validation of DHFR Targeting Through MM-PBSA Calculations

To confirm our hypothesis that spiroacridine **AMTAC-01** overcomes resistance against *qm*DHFR, we performed a Molecular Mechanics Poisson–Boltzmann Surface Area (MM-PBSA) calculation to provide the binding free energy and per-residue contribution in the binding process. Thus, in the first analysis, **AMTAC-01** showed a better value of free binding energy compared to pyrimethamine (ΔG_bind_ = −68.018 and −50.837 kJ.mol^−1^, respectively), confirming the previous findings, with superior binding energy as well as the ability to overcome the quadruple resistance relative to pyrimethamine (Table 4). In addition, for the best fit of the ligand, it is clear that the van der Waals interactions were critical in the coupling process compared to electrostatic interactions (−160.079 and −47.442 kJ.mol^−1^, respectively), similar to the standard compound pyrimethamine (−112.880 and −12.113 kJ.mol^−1^, respectively), confirming the binding mode analysis from the molecular docking, which pointed out the relevance of hydrophobic interactions. Also, the best SASA energy for **AMTAC-01** compared to pyrimethamine (−19.119 and −14.481 kJ.mol^−1^, respectively) suggested the permanence of the compound in the hydrophobic environment, which indicates the best fit at the binding site and corroborates the ΔG_bind_ values.

Finally, the increase in polar solvation energy for **AMTAC-01** compared to pyrimethamine (158.622 and 88.637 kJ.mol^−1^, respectively) suggested the lowest solvation of the ligand and greater permanence at the binding site, away from water molecules, and once again corroborating the ΔG_bind_ values. Furthermore, the per-residue contribution showed that **AMTAC-01** provided more interactions at the binding site of *qm*DHFR compared to pyrimethamine. In this way, the compound showed the best contribution of energy for the residues Ala^16^, Trp^48^, Cys^50^, Met^55^, Phe^58^, and Ile^112^ (−5.0988, −3.6532, −3.4679, −4.8579, −10.6650, and −4.8427 kJ.mol^−1^, respectively) that can improve binding energy and promote greater potential against *qm*DHFR.

## 3. Discussion

In this work, a repurposing of spiroacridines with antitumoral, antileishmanial, and antifungal potential was performed for a possible antimalarial activity towards *Plasmodium falciparum*, the main agent of this infectious disease, considering both sensitive and resistant strains to conventional antimalarial drugs, such as quinolines and artemisinin derivatives. Drug repurposing has emerged as a valuable strategy to accelerate the discovery of novel therapies, reducing the costs and time associated with traditional drug development. This approach is relevant for parasitic diseases, due to the limited investments and rising resistance of parasites against current treatments. Some studies highlight the potential of drug repositioning for protozoan infections and the cross-application of antitumoral compounds to parasitic targets, considering similar features such as rapid proliferation, altered metabolism, and evasion of host defenses [35,36,37].

Along with the assessment of already existing spiroacridines, new ones with different substituents were submitted to the evaluation of the effect of certain moieties on antimalarial activity. Spiroacridines were successfully synthesized and characterized, through IR and NMR spectroscopy, as well as mass spectrometry. Then, the compounds were initially evaluated against the 3D7-GFP strain, which is susceptible to chloroquine and mefloquine and expresses green fluorescent protein (GFP). The production of this protein increases according to the development of the parasite inside the red blood cell (RBC). In this way, the number of green fluorescent events detected in the FITC channel of the flow cytometer at 488 nm consists of the amount of surviving parasites in the culture [38].

**Figure 8:**
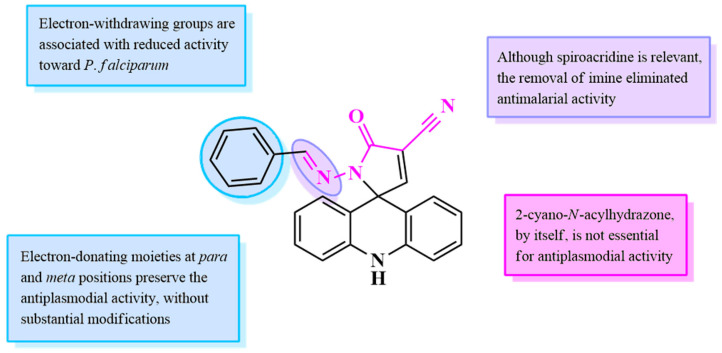
Structure–Activity Relationship (SAR) analysis of spiroacridines for antimalarial activity. Blue color represents modifications in the benzene ring with different substituents or replacement with a heterocycle, whereas pink and purple highlights the relevance or not of 2-cyano-N-acylhydrazone moiety and imine group, respectively.

Considering the substitution profile of compounds (Figure 8), a brief Structure–Activity Relationship (SAR) was performed. From this, it was observed that halogens such as chlorine can reduce the antimalarial potential of this scaffold. However, electron-donating moieties, like methoxy and heterocycles, including quinoline and indole, seem to preserve the activity of the unsubstituted analog. However, the exchange of methoxy with hydroxy at the *para*-position was associated with reduced potential against *P*. *falciparum* 3D7-GFP.

In addition, since the *N*-acylhydrazone intermediates (**JRs**) were inactive against malaria due to inhibition results below 20%, it is possible to suggest that only the *N*-acylhydrazone moiety is not relevant for activity against *P*. *falciparum*. In addition, the spiroacridine alone was also inactive towards malaria, as the removal of the imine group, by exchanging the hydrazine of **AMTAC-01** for the amide of **ACMD-01**, almost completely removed the antimalarial activity of the former. Thus, it can be inferred that both spiroacridine and *N*-acylhydrazone moieties alone do not have antiplasmodial potential, but their association resulted in notable activity against *P*. *falciparum*. After this, the five compounds which showed considerable antiplasmodial activity, with inhibition over 70% at 10 μM, were also active against chloroquine- and artemisinin-resistant strains, showing IC50 values of approximately 2.0 to 4.0 μM.

Considering this and the high values of RI for the standard drug (11.0 and 7.9), as expected for these resistant strains [39], the spiroacridines do not exhibit cross-resistance with quinolines and artemisinins, the main antimalarials used in current treatments [5]. These findings also suggest a possible mechanism of action targeting a different signaling pathway in the parasite compared to both antimalarial drug classes mentioned before. In addition, some antiplasmodial drugs are less potent against *P*. *falciparum* 3D7 compared to chloroquine, such as tafenoquine (1.7 μM) [30], primaquine (9.4 μM) [40], and deferoxamine (27.6 μM) [41], showing that spiroacridines are still promising, with similar potency as antimalarial drugs.

Moreover, the Japanese Global Health Innovative Technology (GHIT) Fund, assembled with experts, established the relevant criteria for the discovery of hit compounds against infectious diseases. It was verified that it is essential for a hit compound to present a selectivity index of at least 10, comparing the activity against parasites with toxicity towards mammalian cells [42]. All compounds evaluated in this work, including chloroquine, showed low cytotoxic effect, with high selectivity indexes over 20 for all parasite strains.

Along with these results obtained in this study, Silva and colleagues (2019) [16] evaluated the acute non-clinical toxicity of **AMTAC-17** in mice, which led to an estimated Median Lethal Dose (LD_50_) greater than 5000 mg/kg, according to OECD guidelines, showing the *in vivo* safety of spiroacridines. In addition to that, Albino and coworkers (2025) [22] evaluated the half-maximal hemolytic concentration (HC_50_) of **AMTAC-01** and **AMTAC-02**, observing no cytotoxicity against erythrocytes up to 400 μM, which also indicates that the antiplasmodial mechanism of action is not correlated with toxicity.

After confirming the antiplasmodial potential of the compounds towards resistant strains and the selectivity of the active spiroacridines, this research aimed to assess their possible *in silico* mechanism of antimalarial action. In this way, the parasite’s DNA synthesis and replication, essential for its development and growth, are associated with several enzymes, including DHFR-TS, LDH, Topo II, and PNPase. Other enzymes, such as ProRS and falcipains, are also crucial for parasite survival through their involvement in protein synthesis. Among these relevant targets, the most probable to justify the antiplasmodial activity of spiroacridines was DHFR-TS, which presented higher affinity scores than the standard drug pyrimethamine in molecular docking simulations. This enzyme is responsible for both folate and thymidylate production, crucial for the synthesis of pyrimidines, purines, and some amino acids involved in the synthesis of DNA. This pathway is relevant for selectivity, considering its absence in humans [34].

To validate the molecular docking and confirm the proposed target, MD simulations were performed against *wt*DHFR and *qm*DHFR. Furthermore, this study also likely generated insights into overcoming DHFR’s resistance to pyrimethamine [43,44,45]. Several mutations are related to the resistance of DHFR to pyrimethamine. The best-known ones are the single mutant S108N, the least resistant; the moderately resistant double mutants such as N51I + S108N and C59R + S108N; as well as the highly resistant triple mutant C59R + S108N + I164L and quadruple-mutant N51I + C59R + S108N + I164L [46]. In this way, our work, in addition to proposing the main drug target of the compounds, focused on providing insights into overcoming DHFR resistance.

After analyzing the 100 ns MD simulation trajectory of both *wt*DHFR and *qm*DHFR enzymes, it is clear that **AMTAC-01** was able to form a stable complex similar to pyrimethamine, and that both compounds showed stability similar to the free protein. On the other hand, in the simulation with *qm*DHFR, as expected, pyrimethamine lost some of its stability, while **AMTAC-01** maintained the stability of the complex, comparable to the free protein. Similar to other works [47,48], MD simulations were crucial to identify critical insights that can help to overcome resistance and design new drugs.

Thus, considering the current status of malaria in the world, with about 263 million cases and 597,000 deaths in 2023 according to WHO, and the growing resistance of *Plasmodium falciparum* against current drugs, the main findings of spiroacridines overcoming resistance *in vitro* against chloroquine and artemisinin drugs, as well as *qm*DHFR towards pyrimethamine, are relevant for the search for new chemotypes targeting resistant parasites, which aligns with WHO’s call for diversification of the antimalarial pipeline [4].

In addition, the analysis of RMSD, RMSF, R_g_, and SASA results supported our findings. The RMSD provides information about deviations that occur in a trajectory, in which smaller deviations indicate better stability of the complex, corroborating the affinity of the compound for the target. In addition, RMSF shows the fluctuations of the residues that provide the permanence of the ligand at the binding site throughout a trajectory [49]. The R_g_ can be used to determine the minor changes during the trajectory that contribute to better rigidity and protein compaction, suggesting stability of the complex formed [50]. As for the SASA, it can be used to investigate the interference of water molecules during the trajectory, in which minor variations, similar to RMSD, RMSF, and R_g_, contribute to the stability of the complex [51]. Another critical experiment was the H-bond simulation [52], in which the simulation with *qm*DHFR demonstrated a decrease in H-bonds to pyrimethamine and an increase in these interactions when simulated with **AMTAC-01**.

Finally, the MM-PBSA results were critical to provide the binding free energy of a ligand in a trajectory of MD simulation and show its affinity against a target [53]. Thus, it was demonstrated that **AMTAC-01** presents a higher binding free energy when complexed with *qm*DHFR compared to the complex with pyrimethamine, and the energetic per-residue contribution highlights more interactions with critical residues of the binding site that contribute to the great potential of **AMTAC-01**. All these data suggest the validation of the protocol, since pyrimethamine resistance is well known, and support the proposal that spiroacridine **AMTAC-01** was able to demonstrate DHFR as a primary target with additional capacity to overcome resistance when simulated with the quadruple-mutant. However, further *in vitro* and *in vivo* validation are required to confirm the DHFR inhibition mechanism suggested by computational models, as well as to fully assess pharmacokinetic properties and systemic toxicity.

## 4. Materials and Methods

### 4.1. Synthesis and Structural Characterization

The reagents applied for the synthesis of the compounds were obtained from Sigma Aldrich, (St. Louis, MO, USA) and all solvents used in the reactions were acquired from Sigma Aldrich, Merck (Darmstadt, Germany), and Fluka (Buchs, Switzerland). To monitor the reactions, Thin-Layer Chromatography (TLC) was carried out with Fluka Analytical silica gel, with 0.2 mm thickness, associated with UV light (250 nm). As for the chemical structure elucidation, Nuclear Magnetic Resonance spectra of ^1^H and ^13^C were acquired with Bruker (80 MHz, Bruker BioSpin, Billerica, MA, USA), Varian Plus (Varian, Santa Clara, CA, USA, 300, 75 MHz), and Bruker Avance (400, 100 MHz, Bruker BioSpin, Billerica, MA, USA), using DMSO-*d*6 as solvent and tetramethylsilane (TMS) as the standard. Chemical shifts were reported in parts per million (ppm), and the multiplicities were presented as follows: singlet (s), doublet (d), doublet of doublet (dd), ddd (doublet of doublet of doublets), t (triplet), td (triplet of doublet), and multiplet (m). In addition, infrared spectroscopy was carried out through two types of techniques: with KBr disks on an IRPrestige-21 spectrometer (Shimadzu^®^, Kyoto, Japan) and by Attenuated Total Reflectance (ATR) on an IRSpirit spectrometer (Shimadzu^®^, Kyoto, Japan). Finally, two different mass spectrometry assays were performed with a high-resolution electrospray ionization mass (HRESIMS–microTOF) and Matrix-Assisted Laser Desorption/Ionization Time-of-Flight (MALDI-TOF) spectrometer (Bruker^®^), in positive ion mode (M + H^+^).

### 4.2. General Procedure for the Synthesis of Spiroacridines

Initially, for the synthesis of *N*-acylhydrazone spiroacridines, the **JR intermediates** were obtained with different substituents, including H (**a**), 4-OCH_3_ (**b**), 4-Cl (**c**), 3,4,5-OCH_3_ (**d**), 4-Quinoline (**e**), 3-Indole (**f**), and 4-OH-3,5-OCH_3_ (**g**). For this, the protocol performed by Ramos et al. (2022) [26] was followed through acidic aldol condensations between 2-cyanoacetohydrazide and different benzaldehydes. Subsequently, these compounds were submitted to Knoevenagel condensation with acridine aldehyde to generate the spiroacridines (**AMTAC**), following the same reaction conditions of the previous study by de Almeida et al. (2016), with ethanol as solvent and triethylamine as base, under reflux [12]. As for the synthesis of acetamide **ACMD-01**, the intermediate 2-cyano-*N*-phenylacetamide was obtained through amidation reaction, following the procedure used by Albino et al. (2025) [22].

For chemical characterization of compounds, the structures of **JR-09**, **JR-18**, **JR-19**, **AMTAC-01**, **AMTAC-02**, **AMTAC-06, AMTAC-17,** and **ACMD-01** were already elucidated in previous studies [12,16,22,25,26,27]. As for the other compounds, their structural elucidation is described below.

**(*E*)-*N*’-(4-chlorobenzylidene)-2-cyanoacetohydrazide (JR-06):** white powder. Molecular formula: C_10_H_8_ClN_3_O. Molecular weight: 221.6430 g/mol. Yield: 85.81%. ^1^H-NMR (DMSO-*d*_6_, 80 MHz). *δ*_ppm_: 11.84 (s, 1H, NH), 8.16 (s, 1H, N=CH conformer), 7.99 (d, 1H, J = 8.0, N=CH), 7.63 (m, 4H, Ar-H), 4.21 (s, 2H, CH_2_), and 3.81 (s, 1H, CH_2_ conformer). IR (ATR, γ, cm^−1^): 3185 (N-H amide), 3095 (C-H sp^2^), 2967 (C-H sp^3^), 2261 (nitrile), 1671 (C=O amide, C=N), 1593 (N-H amide, C=C Ar), 1486 (C=C Ar), 1254 (C-N), 1082 (C-Cl Ar), and 821 (C-H *p*-Ar).

**(*E*)-2-cyano-*N*’-(3,4,5-trimethoxybenzylidene)acetohydrazide (JR-10):** yellow powder. Molecular formula: C_13_H_15_N_3_O_4_. Molecular weight: 277.2759 g/mol. Yield: 95.96%. ^1^H NMR (DMSO-*d*_6_, 80 MHz). *δ*_ppm_: 11.79 (s, 1H, NH), 8.07 (s, 1H, N=CH conformer), 7.91 (s, 1H, N=CH), 7.02 (s, 2H, Ar-H), 4.23 (s, 2H, CH_2_), 3.81 (s, 8H, O-CH_3_), and 3.68 (s, 3H, O-CH_3_). IR (ATR, γ, cm^−1^): 3187 (N-H amide), 3089 (C-H sp^2^), 2994 (C-H sp^3^), 2262 (nitrile), 1677 (C=O amide, C=N), 1580 (N-H amide, C=C Ar), 1501 (C=C Ar), 1234 (C-N, C-O aryl ether), 1127 (C-O aryl ether), 818 (C-H *p*-Ar), 761 (C-H *m*-Ar), and 682 (C-H *m*-Ar).

**(*E*)-2-cyano-*N*’-(4-methoxybenzylidene)acetohydrazide (JR-11):** white powder. Molecular formula: C_11_H_11_N_3_O_2_. Molecular weight: 217.2239 g/mol. Yield: 94.00%. ^1^H NMR (DMSO-*d*_6_, 80 MHz). *δ*_ppm_: 11.65 (s, 1H, NH), 8.09 (s, 1H, N=CH conformer), 7.94 (s, 1H, N=CH), 7.64 (d, J = 8.2 Hz, 2H, benzene), 6.98 (d, J = 8.4 Hz, 2H, benzene), 4.17 (s, 2H, CH_2_), and 3.79 (s, 3H, O-CH_3_). IR (ATR, γ, cm^−1^): 3215 (N-H amide), 3073 (C-H sp^2^), 2966 (C-H sp^3^), 2256 (nitrile), 1671 (C=O amide, C=N), 1605 (C=C Ar), 1566 (N-H amide, 1513 (C=C Ar), 1253 (C-N, C-O aryl ether), 1026 (C-O aryl ether), and 835 (C-H *p*-Ar).

**(*E*)-2-cyano-*N*’-(4-hydroxy-3,5-dimethoxybenzylidene)acetohydrazide (JR-28):** yellow powder. Molecular formula: C_12_H_13_N_3_O_4_. Molecular weight: 263.2493 g/mol. Yield: 84.28%. ^1^H NMR (DMSO-*d*_6_, 400 MHz). *δ*_ppm_: 11.68 (s, 1H, NH), 11.55 (s, 1H, NH conformer), 8.94 (s, 1H, C=CH conformer), 8.88 (s, 1H, C=CH), 8.03 (s, 1H, OH conformer), 7.87 (s, 1H, OH), 6.98 (s, 1H, benzene conformer), 6.97 (s, 1H, benzene conformer), 4.21 (s, 2H, CH_2_), 3.80 (d, J = 3.1 Hz, 2H, O-CH_3_ conformer), 3.79 (d, J = 3.1 Hz, 2H, OCH_3_). ^13^C NMR (DMSO-*d*6, 100 MHz), *δ*_ppm_: 164.54, 158.54, 148.44, 144.86, 148.13, 148.11, 138.22, 137.94, 124.08, 123.99, 116.25, 115.87, 104.86, 104.72, 56.08, 56.07, 24.80, and 24.31. IR (ATR, γ, cm^−1^): 3320 (O-H); 3255 (N-H amide), 3165 (C-H sp^2^), 2958 (C-H sp^3^); 2259 (nitrile), 1684 (C=O amide, C=N), 1595 (C=C Ar), 1517 (N-H amide), 1488 (C=C Ar), 1243 (C-N, C-O aryl ether), 1105 (C-O aryl ether), 835 (C-H *p*-Ar), 741 (C-H *m*-Ar), and 675 (C-H *m*-Ar). HRESIMS: *m/z* 264.0977 [M+H]^+^ (calc. for C_12_H_14_N_3_O_4_, 264.0979).

**(*E*)-5′-oxo-1′-((quinolin-4-ylmethylene)amino)-1′,5′-dihydro-10*H*-spiro[acridine-9,2′-pyrrole]-4′-carbonitrile (AMTAC-21):** yellow powder. Molecular formula: C_27_H_17_N_5_O. Molecular weight = 427.4568 g/mol. Yield: 90.88%. ^1^H NMR (DMSO-*d*_6_, 300 MHz). *δ*_ppm_: 10.10 (s, 1H, NH), 8.80 (d, 1H, quinoline), 8.74 (s, 1H, C=CH), 8.59 (s, 1H, N=CH), 7.97 (dd, 1H, quinoline), 7.87 (dd, 1H, quinoline), 7.73 (m, 1H, quinoline), 7.53 (m, 2H, quinoline), 7.30 (m, 2H, spiroacridine), 7.08 (m, 4H, spiroacridine), and 6.87 (m, 2H, spiroacridine). ^13^C NMR (DMSO-*d*_6_, 75 MHz). *δ*_ppm_: 161.40, 150.65, 148.36, 143.19, 138.99, 137.03, 131.03, 130.21, 129.99, 128.13, 127.71, 124.66, 123.55, 121.22, 119.95, 115.65, 112.51, 111.25, 109.32, and 69.29. IR (ATR, γ, cm^−1^): 3362 (N-H amide), 2240 (nitrile), 1702 (C=O amide, C=N), 1611 (C=C Ar), 1485 (C=C Ar), and 1256 (C-N). MALDI-TOF MS: *m/z* 427.8930 [M + H]^+^ (calc. for C_27_H_17_N_5_O, 427.4568).

**(*E*)-1′-(((1*H*-indol-3-yl)methylene)amino)-5′-oxo-1′,5′-dihydro-10*H*-spiro[acridine-9,2′-pyrrole]-4′-carbonitrile (AMTAC-22):** yellow powder. Molecular formula: C_26_H_17_N_5_O. Molecular weight = 415.4461 g/mol. Yield: 69.84%. ^1^H NMR (DMSO-*d*_6_, 300 MHz). *δ*_ppm_: 11.49 (s, 1H, NH), 9.79 (s, 1H, NH), 8.93 (s, 1H, C=CH), 8.45 (s, 1H, N=CH), 7.62 (m, 2H, indole), 7.34 (m, 1H, indole), 7.26 (m, 2H, indole), 7.11 (m, 2H, spiroacridine), 6.99 (m, 4H, spiroacridine), and 6.85 (m, 2H, spiroacridine). ^13^C NMR (DMSO-*d*_6_, 75 MHz). *δ*_ppm_: 160.80, 159.92, 148.85, 139.26, 137.26, 131.72, 130.29, 127.39, 124.19, 123.18, 122.18, 121.07, 120.67, 115.44, 112.91, 112.62, 112.21, 111.80, 110.02, and 69.72. IR (ATR, γ, cm^−1^): 3453 (NH, amine), 3350 (N-H amide), 3058 (C-H sp^2^), 2241 (nitrile), 1702 (C=O amide, C=N), 1611 (C=C Ar), 1519 (N-H amide, amine), 1479 (C=C Ar), and 1245 (C-N). MALDI-TOF MS: *m/z* 415.606 [M + H]^+^ (calc. for C_26_H_17_N_5_O, 415.4461).

**(*E*)-1′-((4-hydroxy-3,5-dimethoxybenzylidene)amino)-5′-oxo-1′,5′-dihydro-10*H*-spiro[acridine-9,2′-pyrrole]-4′-carbonitrile (AMTAC-24):** yellow powder. Molecular formula: C_26_H_20_N_4_O_4_. Molecular weight = 452.4614 g/mol. Yield: 77.87%. ^1^H NMR (DMSO-*d*_6_, 400 MHz). *δ*_ppm_: 9.85 (s, 1H, NH), 8.57 (s, 1H, C=CH), 8.32 (s, 1H, N=CH), 7.28 (m, 2H, spiroacridine), 7.02 (ddd, J_1_ = 14.55 Hz, J_2_ = 8.09 Hz, J_3_ = 1.37 Hz, 4H, spiroacridine), 6.86 (td, J_1_ = 8.07 Hz, J_2_ = 7.12 Hz, J_3_ = 1.20 Hz, 2H, spiroacridine), 6.66 (s; 2H, benzene), and 3.69 (s; 6H, O-CH_3_). ^13^C NMR (DMSO-*d*_6_, 100 MHz). *δ*_ppm_: 160.73, 160.05, 148.71, 148.04, 138.86, 138.55, 130.11, 126.96, 124.03, 120.40, 115.04, 112.46, 111.87, 108.86, 104.66, 68.96, 56.06, and 55.94. IR (ATR, γ, cm^−1^): 3534 (O-H phenol), 3311 (N-H amide), 3085 (C-H sp^2^), 2924 (C-H sp^3^), 2236 (nitrile), 1705 (C=O amide, C=N), 1612 (C=C Ar), 1586 (N-H amide), 1483 (C=C Ar), 1222 (C-N, C-O aryl ether), 1115 (C-O aryl ether), 831 (C-H *p*-Ar), 744 (C-H *m*-Ar), and 660 (C-H *m*-Ar). HRESIMS: *m/z* 453.1546 [M + H]^+^ (calc. for C_26_H_21_N_4_O_4_, 453.1557).

### 4.3. Investigation of Antiplasmodial Activity Against Asexual Blood Stages

Firstly, to prepare the samples for *in vitro* assays, each compound was dissolved in DMSO (Sigma-Aldrich^®^, Merck, Darmstadt, Germany), obtaining a solution of 5 mM, from which two other solutions, at 100 μM and 10 μΜ were prepared through dilution with RPMI-1640 medium (Invitrogen™, Carlsbad, CA, USA), supplemented with AlbuMAXII (Invitrogen™, Carlsbad, CA, USA). In parallel, the parasite cultures used in the *in vitro* assays include the following *P*. *falciparum* strains: 3D7-GFP (MRA-1029, MR4, ATCC^®^, Manassas, VA, USA, chloroquine-sensitive, expressing Green Fluorescent Protein), Dd2 (MRA-150, ATCC^®^, Manassas, Virgínia, chloroquine-resistant), and IPC5202 (MRA-1240, ATCC^®^, Manassas, VA, USA, artemisinin-resistant). These cultures were obtained through BEI Resources, NIAID, NIH (MR4, ATCC^®^, Manassas, VA, USA), contributed by Andrew M. Talman, Robert E. Sinden, David Walliker, and Didier Ménard. The parasites were cultivated with 5% hematocrit at 37 °C and an atmosphere enriched with 5% of CO_2_ [54].

Then, to assess the antiplasmodial activity of the compounds, a screening assay was initially performed against the *P*. *falciparum* 3D7-GFP strain. For this, unsynchronized cultures were incubated in a 96-well flat-bottom plate at 1.2% hematocrit and 1.0% parasitemia, in the presence and absence of 1 μM and 10 μM of each compound, for 72 h at 37 °C and 5% CO_2_. Chloroquine (Sigma-Aldrich^®^, Merck, Darmstadt, Germany) was used as a standard drug at the same concentrations as the other compounds. Parasite growth was determined by flow cytometry (CytoFLEX, Beckman Coulter Life Sciences, Indianapolis, IN, USA) with a 96-well plate reader, using Fl-1 with an excitation wavelength of 488 nm for GFP detection. The flow cytometry data were analyzed with FlowJo v10 software (FlowJo LLC, Ashland, OR, USA) to calculate the percentage inhibition of compounds.

The most potent compounds in the screening, with at least 70% inhibition at the highest concentration (10 µM), were selected for further development by the determination of half-maximal inhibitory concentrations (IC_50_) [38]. In order to accomplish this, unsynchronized cultures of 3D7-GFP, Dd2, and MRA-1240 were incubated at 1.2% hematocrit and 1.0% parasitemia with serial three-fold dilutions of the compounds, ranging from 10,000 nM to 0.17 nM, in 96-well flat-bottom plates for 72 h at 37 °C and 5% CO_2_. Parasite growth was quantified by flow cytometry (CytoFLEX, Beckman Coulter Life Sciences, Indianapolis, IN, USA) using GFP detection in a 96-well plate reader (Fl-1 channel) for the 3D7-GFP strain. The others (Dd2 and MRA-1240) were stained with SYBR Green I (20×), which is also detected at 488 nm. Data analysis was performed with FlowJo v10 software (FlowJo LLC, Ashland, OR, USA), and the half-maximal inhibitory concentrations (IC_50_) were calculated using GraphPad Prism 5 (trial version), with mean IC_50_ values obtained from at least two independent experiments, each conducted in triplicate.

### 4.4. Evaluation of Cytotoxicity Against Mammalian Cells

In order to evaluate the cytotoxicity of the compounds, a Vero E6 viability assay (3-(4,5-dimethylthiazol-2-yl)-2,5-diphenyltetrazolium bromide) was performed only for the active compounds which passed through the antiplasmodial screening and were submitted to the IC_50_ assay. For this, monkey kidney cells (Vero E6) were seeded at a density of 5 × 10^3^ cells/mL in 96-well plates and allowed to adhere for 6 h. Vero E6 cell line was obtained from the Cell Bank of Rio de Janeiro and cultured in DMEM media (Dulbecco’s Modified Eagle Medium) plus 10% inactivated fetal bovine serum (FBS) and 1% penicillin–streptomycin. Treatments were performed by adding 100 μL of medium solutions with test drugs, resulting in concentrations ranging from 12.5 to 100 µM (DMSO 1%, *v*/*v*). After 72 h of incubation at 37 °C and under 5% of CO_2_, the cultures were incubated with MTT at 0.5 mg/mL for 2 h. After this, the formazan crystals were dried in room temperature and dissolved in DMSO. Cell viability was determined by measuring the absorbance at 570 nm (Absorbance microplate reader EL800, BioTek, Winooski, VT, USA), and control wells containing DMSO at 1% were considered as 100% cell viability [55].

### 4.5. Statistical Analysis

All statistical analyses and graphical representations were performed using the GraphPad Prism 8.0 software package. Dose–response curves were generated by fitting the data to a nonlinear regression model [log(inhibitor) vs. response—variable slope]. Concentration values were logarithmically transformed, and data were normalized so that the minimum and maximum responses corresponded to 0% and 100% inhibition, respectively. The IC_50_ values were obtained from the fitted curves. Each experiment was carried out in triplicate and repeated independently, and results were expressed as mean ± standard deviation.

### 4.6. Molecular Docking

The possible mechanism of antimalarial action was proposed through molecular docking, which was conducted using the most active compounds identified through *in vitro* assays and selected crystallographic structures obtained from the Protein Data Bank. The following targets were evaluated: dihydroorotate dehydrogenase (ID: 9DIK), wild-type and quadruple-mutant bifunctional dihydrofolate reductase-thymidylate synthase (ID: 3QGT and 3QG2), purine nucleoside phosphorylase (ID: 5ZNC), topoisomerase II (ID: 6CA8), prolyl-tRNA synthetase (ID: 4WI1), lactate dehydrogenase (ID: 1U4O), and falcipains 2 and 3 (ID: 3BPF and 3BWK) of *P. falciparum*. Molecular structures were drawn using MarvinSketch, ChemAxon (https://www.chemaxon.com), and 10 conformations were generated per compound, with the most stable one geometrically optimized with ORCA [56,57], using the semi-empirical PM3 (Parametric Method 3) method [58].

The protein structures were submitted to the Genetic Optimization for Ligand Docking—GOLD 2022.3.0 software [59]—to perform molecular docking simulations. These were treated with the addition of hydrogens and the removal of water molecules, maintaining the essential cofactors for each target. After that, the active site was selected in a region of 6.0 Å around the co-crystallized ligand, with 10 Genetic Algorithm operations performed for each molecule. For falcipains 2 and 3, cysteine proteases, covalent docking was necessary to promote the formation of a covalent bond between a chemical group of the compound and the Cys^42^ residue of the enzyme. In the case of topoisomerase II*,* due to the absence of a co-crystallized ligand, the active site of the enzyme was determined according to the catalytic residues reported in the literature [32].

Subsequently, the conformations of the molecules with the highest Fitness Score were selected for further analysis of the binding regions, types of interactions, and amino acids of the active site that participate in the bonds using Discovery Studio 2021 v21.1.0.20298 software. Next, to validate the methodology, molecular redocking was performed, prioritizing conformations with the lowest RMSD of the distances between the atoms and below 2.0 Å. This protocol followed others published by our research group [50,51,60,61].

### 4.7. Homology Modeling

The 3D structure of *wt*DHFT and *qm*DHFR under the PDB codes 3QGT and 3QG2, respectively, were submitted to the SWISS-MODEL web tool [62] to build the complete structure of the target to use in the MD simulations. Thus, the FASTA sequence of both PDBs was added to the SWISS-MODEL to search for similar templates. In this way, the best template for *wt*DHFT and *qm*DHFR was the bifunctional dihydrofolate reductase-thymidylate synthase (Code: Q8I1R6.1), available in AlphaFold DB (gene: Q8I1R6_PLAF7) from *Plasmodium falciparum* (3D7). Finally, the 3D structure of both targets was built and used in MD simulations.

### 4.8. Molecular Dynamics (MDs) Simulations

To confirm the findings from molecular docking, MD simulations were performed with the most promising target DHFR, wild-type and quadruple-mutant, in complex with the most active compound (**AMTAC-01)**. In addition, the MD simulations were performed using the *wt*DHFR and *qm*DHFR structures constructed by homology modeling, as they represent the complete structure of the target. Thus, the MD simulations were performed using the GROMACS 2020^®^ software. Initially, charges and hydrogens were added using UCSF Chimera^®^ 1.17.1 software through the DockPrep tool. Then, the CHARMM36 force field was applied using the TIP3P solvation method. In parallel, ligand topology was generated using the web software SwissParam (http://www.swissparam.ch/) [63]. Next, a 1.0 nm triclinic box was created, adding water and ions at physiological concentration. This was followed by system equilibrium in 10,000 steps by the conjugate gradient method and the system’s total minimization in 20,000 steps. After this, NVT (constant number of particles, volume, and temperature) and NPT (constant number of particles, pressure, and temperature) equilibriums were performed at 300 K for 10 ns. The final simulation was performed at 100 ns. The Root Mean Square Deviation (RMSD), Root Mean Square Fluctuation (RMSF), radius of gyration (R_g_), and Solvent Accessible Surface Area (SASA) plots were generated using the Xmgrace^®^ 5.1.25 software. This protocol agrees with other previously published works from our research team [49,50,51,52,61,64,65].

### 4.9. MM-PBSA Calculations

The Molecular Mechanics Poisson–Boltzmann Surface Area (MM-PBSA) method was used to calculate the Gibbs free binding energy (ΔG_binding_) based on van der Waals and electrostatic (unbound) interactions between the ligand and its receptor during an MD simulation. For this, Δ*G*_binding_ was calculated as the difference between the free energy of the protein–ligand complex (*G*_complex_) and the unbound protein and ligand (*G*_protein_ and *G*_ligand_) (Equation (1)). These individual energy values are calculated from the average potential energy of molecular mechanics in vacuum (*E*_MM_) minus the entropic contribution to the free energy in vacuum by the temperature and entropy (TS) added to the solvation energy (*G*_solvation_) (Equation (2)). Furthermore, *E*_MM_ is the sum of bonded interactions such as dihedral, angle, bond, and improper (*E*_bond_), and non-bonded interactions (*E*_non-bonded_), that constitute the electrostatic (*E*_elec_) and van der Waals interactions (*E*_vdw_) using the potential functions of Coulomb and Lennard-Jones, respectively (Equation (3)). Finally, the solvation free energy (*G*_solvation_) is the sum of electrostatic and non-electrostatic contributions in the solvation free energy (*G*_polar_ and *G*_non-polar_, respectively) (Equation (4)). These calculations were performed using the g_mmpbsa tool [66] through the trajectory files obtained after MD simulations using GROMACS 2020^®^ software. Then, ΔG_binding_ values were determined as the average free interaction and solvation energies [67]. This protocol is based on other works from our research group [50,51,52,53].

ΔG_binding_ = G_complex_ − (G_protein_ − G_ligand_) (1)

G_x_ = (E_MM_) − TS + (G_solvation_) (2)

E_MM_ = E_bonded_ + E_non-bonded_ = E_bonded_ + (E_vdw_ + E_elec_) (3)

G_solvation_ = G_polar_ + G_non-polar_ (4)

## 5. Conclusions

Considering the results obtained in this study, spiroacridines were successfully synthesized, and their structure was confirmed by spectroscopy and spectrometry. The spiroacridine derivatives, particularly **AMTAC-01**, exhibited potent *in vitro* antiplasmodial activity against both chloroquine- and artemisinin-resistant *P. falciparum* strains, with low cytotoxicity toward mammalian cells (Vero 6). For the mechanism of action, through molecular docking, MD simulations, and MM-PBSA calculations. Our work proposed DHFR as the primary target related to the antimalarial activity of the compounds. Our findings suggest that **AMTAC-01** is capable of overcoming resistance to *qm*DHFR, generating essential insights in medicinal chemistry and drug discovery.

## Figures and Tables

**Table 1:** *In vitro* antimalarial activity against drug-sensitive and drug-resistant *P. falciparum* strains (3D7-GFP, Dd2, and MRA-1240) after 72 h of treatment.

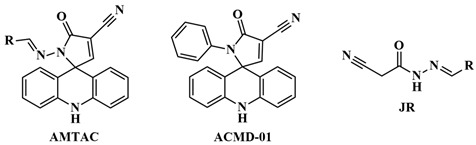							
Compound	R	Inhibition (%)	IC_50_ (μM) ^b^			RI ^c^	
		10 μΜ	3D7-GFP	Dd2	MRA-1240	Dd2	MRA-1240
**AMTAC-01**	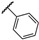	96.8 ± 0.1	2.11 ± 0.60	1.37 ± 0.46	1.66 ± 0.08	0.6	0.8
**AMTAC-02**	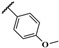	97.6 ± 0.1	3.19 ± 0.49	3.12 ± 0.34	4.01 ± 0.17	1.0	1.3
**AMTAC-06**	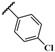	62.4 ± 8.9	–	–	–	–	–
**AMTAC-17**	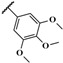	97.4 ± 0.1	3.28 ± 0.49	3.29 ± 0.38	4.09 ± 0.05	1.0	1.2
**AMTAC-21**	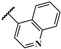	91.1 ± 0.1	3.78 ± 0.18	3.99 ± 0.23	4.07 ± 0.27	1.0	1.1
**AMTAC-22**	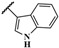	95.3 ± 0.1	3.01 ± 0.52	2.34 ± 0.11	2.88 ± 0.30	0.8	1.0
**AMTAC-24**	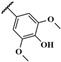	66.8 ± 10.8	–	–	–	–	–
**ACMD-01**	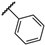	9.8 ± 1.6	–	–	–	–	–
**JR-06**	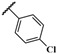	12.4 ± 1.0	–	–	–	–	–
**JR-09**	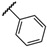	14.8 ± 0.4	–	–	–	–	–
**JR-10**	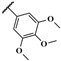	12.6 ± 1.4	–	–	–	–	–
**JR-11**	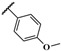	13.4 ± 1.4	–	–	–	–	–
**JR-18**	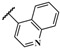	14.7 ± 7.2	–	–	–	–	–
**JR-19**	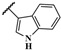	16.8 ± 5.0	–	–	–	–	–
**JR-28**	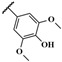	5.5 ± 11.5	–	–	–	–	–
**CQ ^a^**	–	94.34 ± 0.54	0.02 ± 0.01	0.26 ± 0.09	0.19 ± 0.07	11.0	7.9

**^a^** CQ = chloroquine. ^b^ Half-Maximal Inhibitory Concentration (IC_50_, μM). The results for each compound were calculated relative to the control in two independent experiments, each performed in triplicate. Results are presented as mean ± standard deviation (SD). **^c^** RI (resistance index) = IC_50_ (Dd2 or MRA-1240)/IC_50_ (3D7).

**Table 2:** *In vitro* cytotoxicity against monkey kidney cells (Vero E6) after 72 h of treatment.

Compound	IC_50_ (μM) ^b^				SI ^c^		
	3D7-GFP	Dd2	MRA-1240	Vero E6	3D7-GFP	Dd2	MRA-1240
**AMTAC-01**	2.11 ± 0.60	1.37 ± 0.46	1.66 ± 0.08	>100.00	>47.3	>72.7	>60.2
**AMTAC-02**	3.19 ± 0.49	3.12 ± 0.34	4.01 ± 0.17	>100.00	>31.3	>32.0	>24.9
**AMTAC-17**	3.28 ± 0.49	3.29 ± 0.38	4.09 ± 0.05	>100.00	>30.5	>30.4	>24.5
**AMTAC-21**	3.78 ± 0.18	3.99 ± 0.23	4.07 ± 0.27	>100.00	>26.4	>25.1	>24.6
**AMTAC-22**	3.01 ± 0.52	2.34 ± 0.11	2.88 ± 0.30	>100.00	>33.2	>42.7	>34.7
**CQ ^a^**	0.02 ± 0.01	0.26 ± 0.09	0.19 ± 0.07	>100.00	>4251.2	>387.5	>539.2

^a^ CQ = chloroquine. ^b^ Half-Maximal Inhibitory Concentration (IC_50_, μM). The results for each compound were calculated relative to the control in two independent experiments, each performed in triplicate. Results are presented as mean ± standard deviation (SD). **^c^** SI (selectivity index) = IC_50_ (Vero E6)/IC_50_ (3D7-GFP, Dd2 or MRA-1240).

**Table 3:** FitScore values of spiroacridine derivatives against relevant *P. falciparum* targets.

Target	RMSD ^10^ (Å)	FitScore					
		AMTAC-01	AMTAC-02	AMTAC-17	AMTAC-21	AMTAC-22	Standard ^11^
**DHODH ^1^**	0.78	58.22	46.79	13.49	56.60	50.98	74.96
**W-DHFR ^2^**	0.41	70.45	68.33	70.36	71.33	75.16	65.65
**M-DHFR ^3^**	0.46	65.53	61.73	66.78	70.66	72.85	63.45
**PNPase ^4^**	0.45	46.48	49.04	44.08	51.08	49.58	64.76
**Topo II ^5^**	–	41.23	46.61	48.80	40.21	40.35	50.77
**ProRS ^6^**	1.01	48.06	56.75	48.58	53.05	55.58	67.43
**LDH ^7^**	0.51	50.25	49.38	54.95	53.96	54.62	56.94
**FP2 ^8^**	0.73	70.38	74.74	69.08	79.23	74.13	108.76
**FP3 ^9^**	1.08	78.06	72.73	63.90	80.82	74.51	131.94

^1^ *Plasmodium falciparum* dihydroorotate dehydrogenase (ID: 9DIK); ^2^ *Plasmodium falciparum* wild-type bifunctional dihydrofolate reductase-thymidylate synthase (ID: 3QGT); ^3^ *Plasmodium falciparum* quadruple-mutant bifunctional dihydrofolate reductase-thymidylate synthase (ID: 3QG2); ^4^ *Plasmodium falciparum* purine nucleoside phosphorylase (ID: 5ZNC); ^5^ *Plasmodium falciparum* topoisomerase II (ID: 6CA8); ^6^ *Plasmodium falciparum* prolyl-tRNA synthetase (ID: 4WI1); ^7^ *Plasmodium falciparum* lactate dehydrogenase (ID: 1U4O); ^8^ Falcipain-2 (ID: 3BPF); ^9^ Falcipain-3 (ID: 3BWK); ^10^ RMSD: Root Mean Square Deviation; ^11^ DSM681 (DHODH), pyrimethamine (DHFR W and M), quinine (PNPase), etoposide (Topo II), TCMDC-124506 (ProRS), 2,6-naphthalenedicarboxylic acid (LDH), E64 (FP2), and K11017 (FP3).

**Table 4:** Results of binding free energy calculation and interaction parameters by MM-PBSA of the pyrimethamine and **AMTAC-01** in complex with *qm*DHFR.

Compound	ΔG_bind_ (kJ.mol^−1^)	SASA (kJ.mol^−1^)	Polar solvation (kJ.mol^−1^)	Electrostatic (kJ.mol^−1^)	van der Waals (kJ.mol^−1^)
**Pyrimethamine ^1^**	−50.837 ± 10.296	−14.481 ± 0.821	88.637 ± 20.564	−12.113 ± 14.691	−112.880 ± 12.000
**AMTAC-01**	−68.018 ± 13.969	−19.119 ± 1.185	158.622 ± 32.375	−47.442 ± 15.868	−160.079 ± 21.005

^1^ the pyrimethamine used as a standard compound was co-crystallized in PDB 3QG2.

## Data Availability

Data are available in the article and the Supplementary Materials.

## Supplementary Materials

The following supporting information can be downloaded at: https://www.mdpi.com/article/10.3390/antibiotics14121214/s1. Figure S1: ^1^H NMR spectrum of JR-06 (80 MHz, DMSO-*d*_6_; Figure S2: ^1^H NMR spectrum of JR-10 (80 MHz, DMSO-*d*_6_); Figure S3: ^1^H NMR spectrum of JR-11 (80 MHz, DMSO-*d*_6_); Figure S4: ^1^H NMR spectrum of JR-28 (400 MHz, DMSO-*d*_6_); Figure S5: ^13^C NMR spectrum of JR-28 (100 MHz, APT, DMSO-*d*_6_); Figure S6: ^1^H NMR spectrum of AMTAC-21 (400 MHz, DMSO-*d*_6_); Figure S7: ^13^C NMR spectrum of AMTAC-21 (100 MHz, BB, DMSO-*d*_6_); Figure S8: ^1^H NMR spectrum of AMTAC-22 (400 MHz, DMSO-*d*_6_); Figure S9: ^13^C NMR spectrum of AMTAC-22 (100 MHz, BB, DMSO-*d*_6_); Figure S10: ^1^H NMR spectrum of AMTAC-24 (400 MHz, DMSO-*d*_6_); Figure S11: ^13^C NMR spectrum of AMTAC-24 (100 MHz, APT, DMSO-*d*_6_); Figure S12: Infrared spectrum of JR-06 (ATR); Figure S13: Infrared spectrum of JR-10 (ATR); Figure S14: Infrared spectrum of JR-11 (ATR); Figure S15: Infrared spectrum of JR-28 (ATR); Figure S16: Infrared spectrum of AMTAC-21 (ATR); Figure S17: Infrared spectrum of AMTAC-22 (ATR); Figure S18: Infrared spectrum of AMTAC-24 (ATR); Figure S19: Mass spectrum of JR-28 by HRESIMS; Figure S20: Mass spectrum of AMTAC-21 by MALDI-TOF; Figure S21: Mass spectrum of AMTAC-22 by MALDI-TOF; Figure S22: Mass spectrum of AMTAC-24 by HRESIMS; Figure S23: Concentration-response curves of AMTAC compounds and Chloroquine against *Plasmodium falciparum* 3D7-GFP strain; Figure S24: Concentration-response curves of AMTAC compounds and Chloroquine against *Plasmodium falciparum* Dd2 strain; Figure S25: Concentration-response curves of AMTAC compounds and Chloroquine against *Plasmodium falciparum* MRA-1240 strain.

## Abbreviations

The following abbreviations are used in this manuscript:

^1^H-NMR	1-Hydrogen Nuclear Magnetic Resonance
3D7-GFP	*Plasmodium falciparum* 3D7HT-GFP
^13^C-NMR	13-Carbon Nuclear Magnetic Resonance
ΔG_bind_	Gibbs Free Binding Energy
ATR	Attenuated Total Reflectance
CQ	Chloroquine
DHFR	Dihydrofolate Reductase
DHODH	Dihydroorotate Dehydrogenase
ENR	Enoyl Acyl Carrier Protein Reductase
FP2	Falcipain-2
FP3	Falcipain-3
GFP	Green Fluorescent Protein
GHIT	Japanese Global Health Innovative Technology
GOLD	Genetic Optimization for Ligand Docking
HRESIMS	High-Resolution Electrospray Ionization Mass
IC_50_	Half-Maximal Inhibitory Concentration
LD_50_	Median Lethal Dose
LDH	Lactate Dehydrogenase
MALDI-TOF	Matrix-Assisted Laser Desorption/Ionization Time-of-Flight
MD	Molecular Dynamics
MM-PBSA	Molecular Mechanics Poisson–Boltzmann Surface Area
PNPase	Purine Nucleoside Phosphorylase
ProRS	Prolyl-tRNA Synthetase
*qm*DHFR	Quadrupole-Mutated Dihydrofolate Reductase
R_g_	Radius of Gyration
RI	Resistance Index
RMSD	Root Mean Square Deviation
RMSF	Root Mean Square Fluctuation
SAR	Structure–Activity Relationship
SASA	Solvent Accessible Surface Area
SI	Selectivity Index
TLC	Thin-Layer Chromatography
Topo II	Topoisomerase II
*wt*DHFR	Wild-Type Dihydrofolate Reductase
