# Supplementary material for: Design, Synthesis, and Antimalarial Evaluation of New Spiroacridine Derivatives
Source: Antibiotics (Basel). 2025 Dec 2;14(12):1214. doi: 10.3390/antibiotics14121214 (PMC12730060; doi:10.3390/antibiotics14121214)
Supplement: Supplementary file 1 [file antibiotics-14-01214-s001.zip › antibiotics-3933808-supplementary.pdf]

Design, Synthesis, and Antimalarial Evaluation of New Spiroacridine Derivatives

Misael de Azevedo Teotônio Cavalcanti [1,2], Sonaly Lima Albino [1,2], Karla Joane da Silva Menezes [2], Wallyson Junio Santos de Araújo [2], Fernanda de França Genuíno Ramos Campos [2], Malu Maria Lucas dos Reis [2,3], Inês Morais [4], Denise Maria Figueiredo Araújo Duarte [4], Igor José dos Santos Nascimento [1,2], Valnês da Silva Rodrigues-Junior [5], Fátima Nogueira [4,*] and Ricardo Olímpio de Moura [1,2,*]

[1] Programa de Pós-Graduação em Ciências Farmacêuticas (PPGCF), Universidade Estadual da Paraíba (UEPB), Campina Grande 58429-500, PB, Brazil; misaelazevedo.2015@gmail.com (M.d.A.T.C.); sonaly.albino@hotmail.com (S.L.A.); igorjsn@hotmail.com (I.J.d.S.N.)

[2] Laboratório de Desenvolvimento e Síntese de Fármacos (LDSF), Universidade Estadual da Paraíba (UEPB), Campina Grande 58429-500, PB, Brazil; menezeskarla5@gmail.com (K.J.d.S.M.); wallyson.araujo@aluno.uepb.edu.br (W.J.S.d.A.); fernanda.campos@aluno.uepb.edu.br (F.d.F.G.R.C.); malureisduarte@gmail.com (M.M.L.d.R.)

[3] Departamento de Química, Universidade Estadual da Paraíba (UEPB), Campina Grande 58429-500, PB, Brazil

[4] Global Health and Tropical Medicine (GHTM), Associate Laboratory in Translation and Innovation Towards Global Health, LA-REAL, Instituto de Higiene e Medicina Tropical (IHMT), Universidade NOVA de Lisboa (UNL), Rua da Junqueira 100, 1349-008 Lisboa, Portugal; ines.morais@ihmt.unl.pt (I.M.); dduarte@ihmt.unl.pt (D.M.F.A.D.)

[5] Programa de Pós-Graduação em Produtos Naturais e Sintéticos Bioativos (PgPNSB), Departamento de Ciências Farmacêuticas, Universidade Federal da Paraíba (UFPB), João Pessoa 58051-900, PB, Brazil; valnesjunior@cbiotec.ufpb.br

* Correspondence: fnogueira@ihmt.unl.pt (F.N.); ricardo.olimpiodemoura@gmail.com or ricardo.olimpiodemoura@servidor.uepb.edu.br (R.O.d.M.); Tel.: +55-83-99628-0751 (R.O.d.M.)

Supplementary Material

Table of Contents

1. Chemical structure elucidation of compounds .... Figures S1-S22

2. Concentration-response curves .... Figures S23-S25

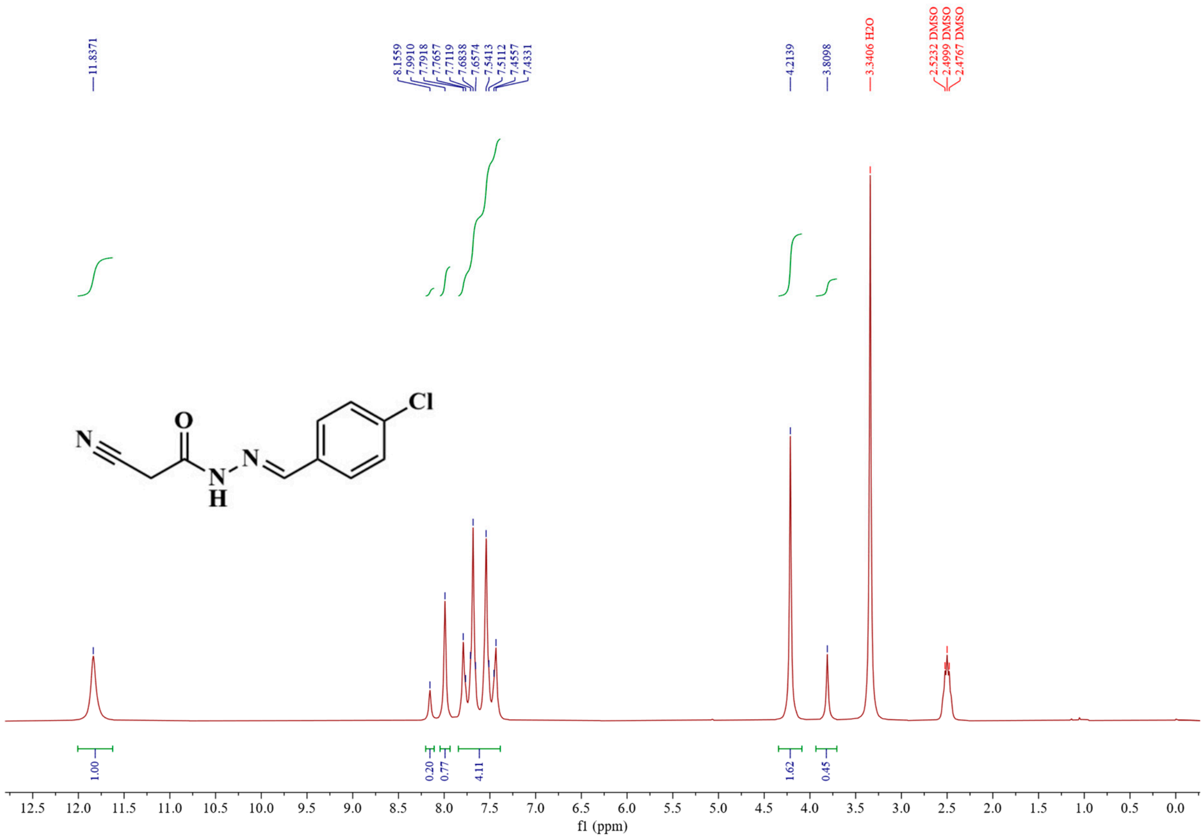

Figure S1. ^{1}H NMR spectrum of JR-06 (80 MHz, DMSO-d_6).

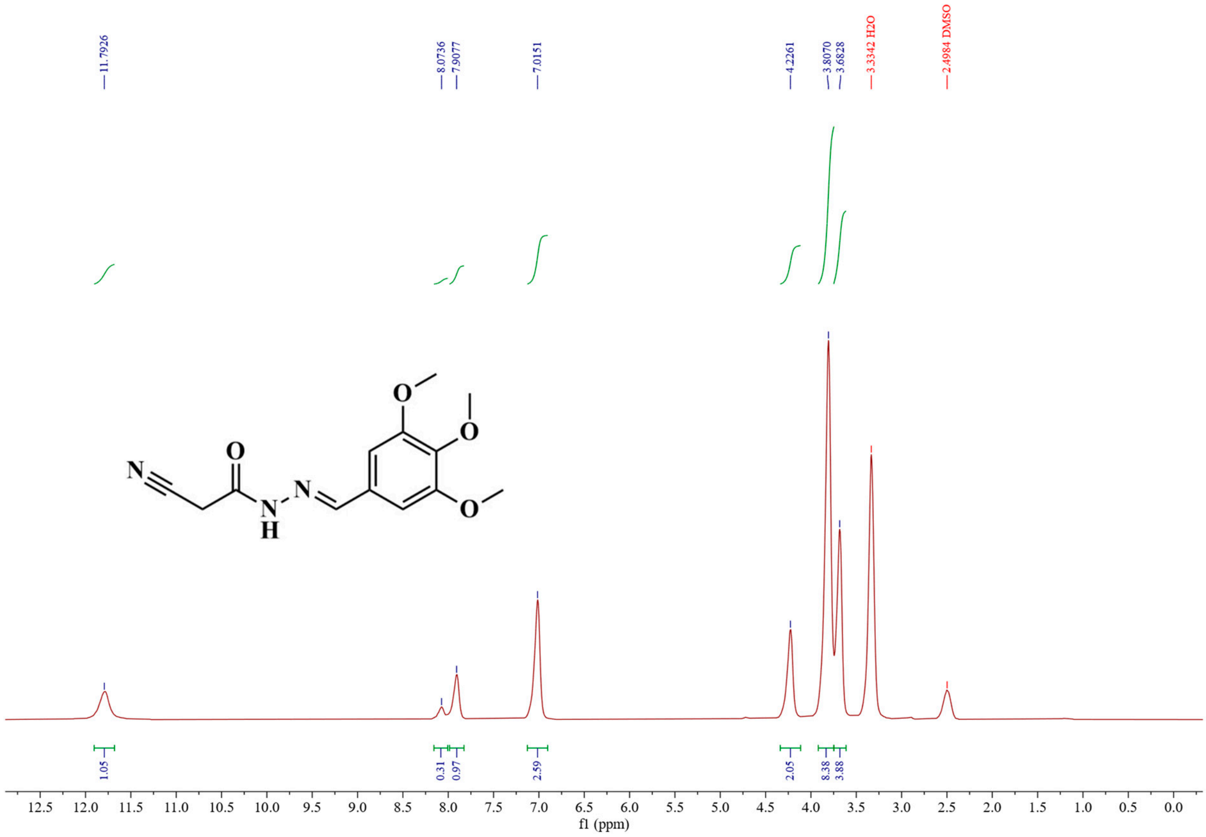

Figure S2. ^{1}H NMR spectrum of JR-10 (80 MHz, DMSO-d_6).

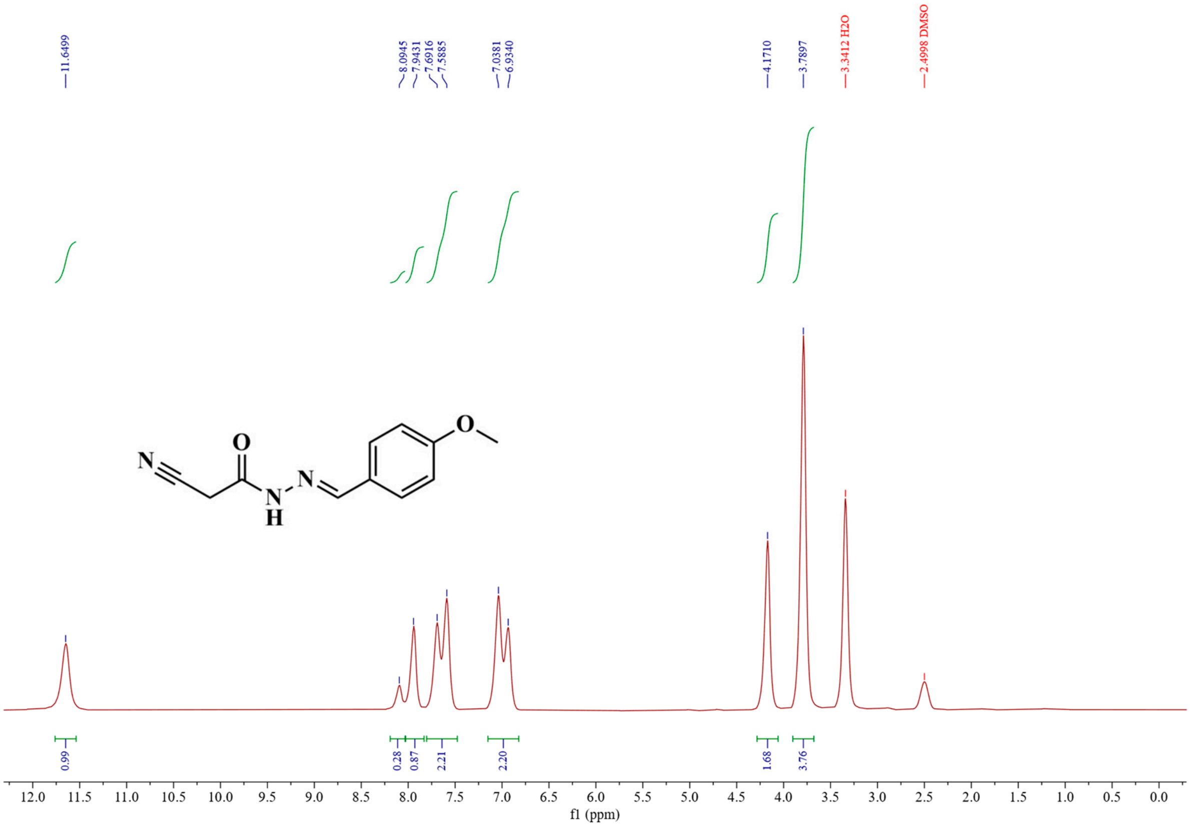

Figure S3. ^{1}H NMR spectrum of JR-11 (80 MHz, DMSO-d_6).

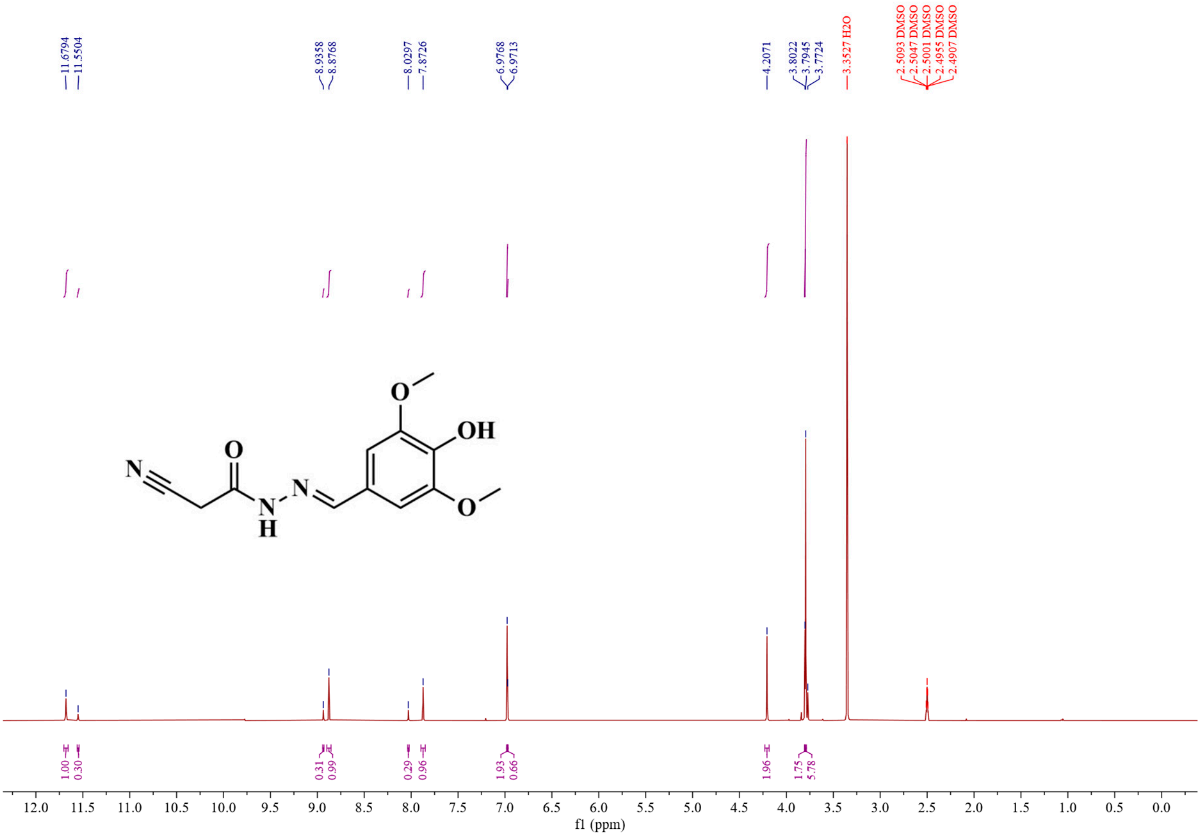

Figure S4. ^{1}H NMR spectrum of JR-28 (400 MHz, DMSO-d_6).

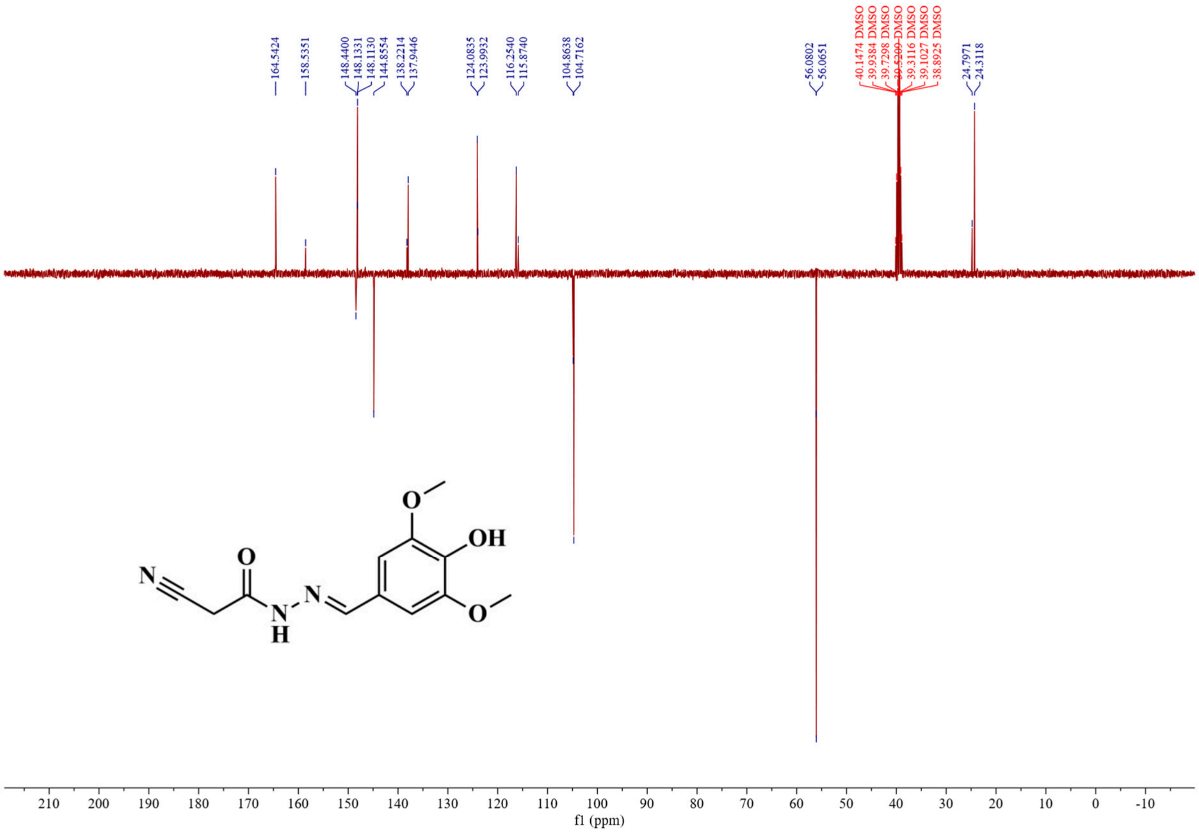

Figure S5. ^{13}C NMR spectrum of JR-28 (100 MHz, APT, DMSO-d_6).

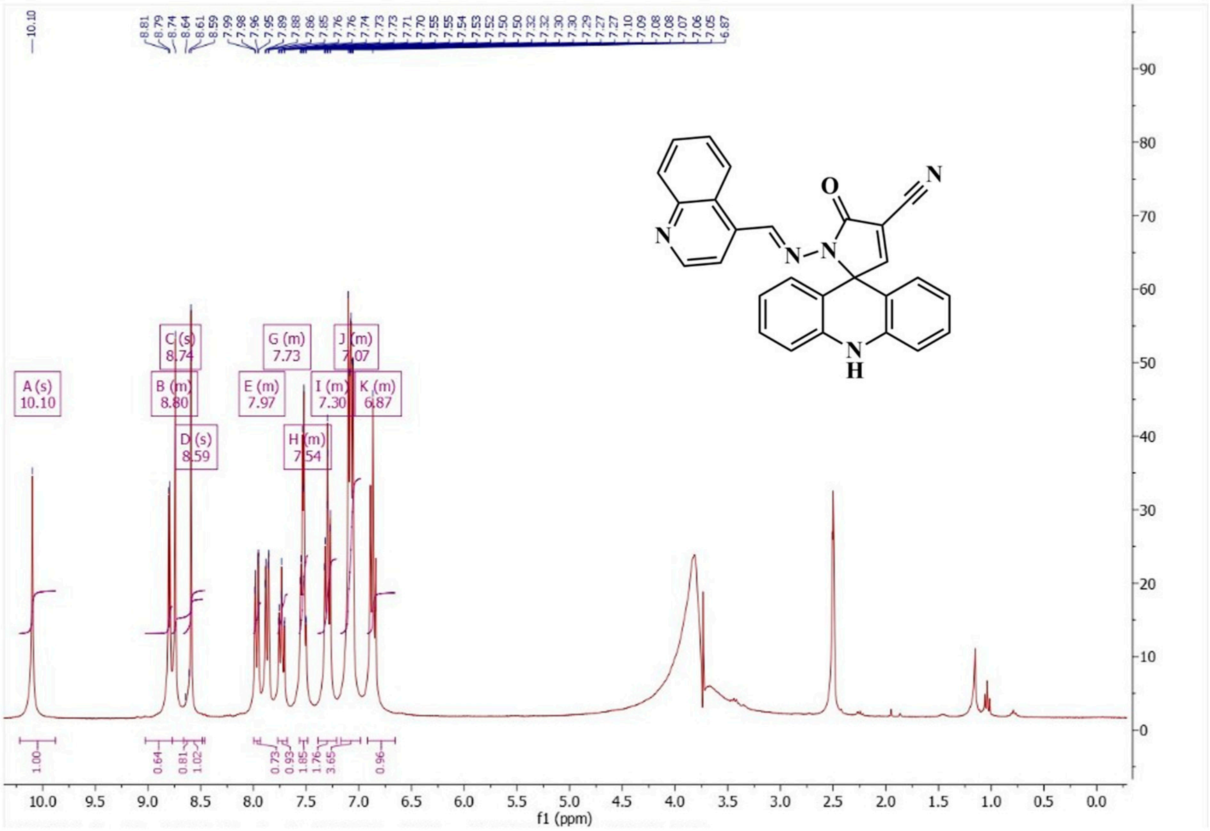

Figure S6. ^{1}H NMR spectrum of AMTAC-21 (400 MHz, DMSO-d_6).

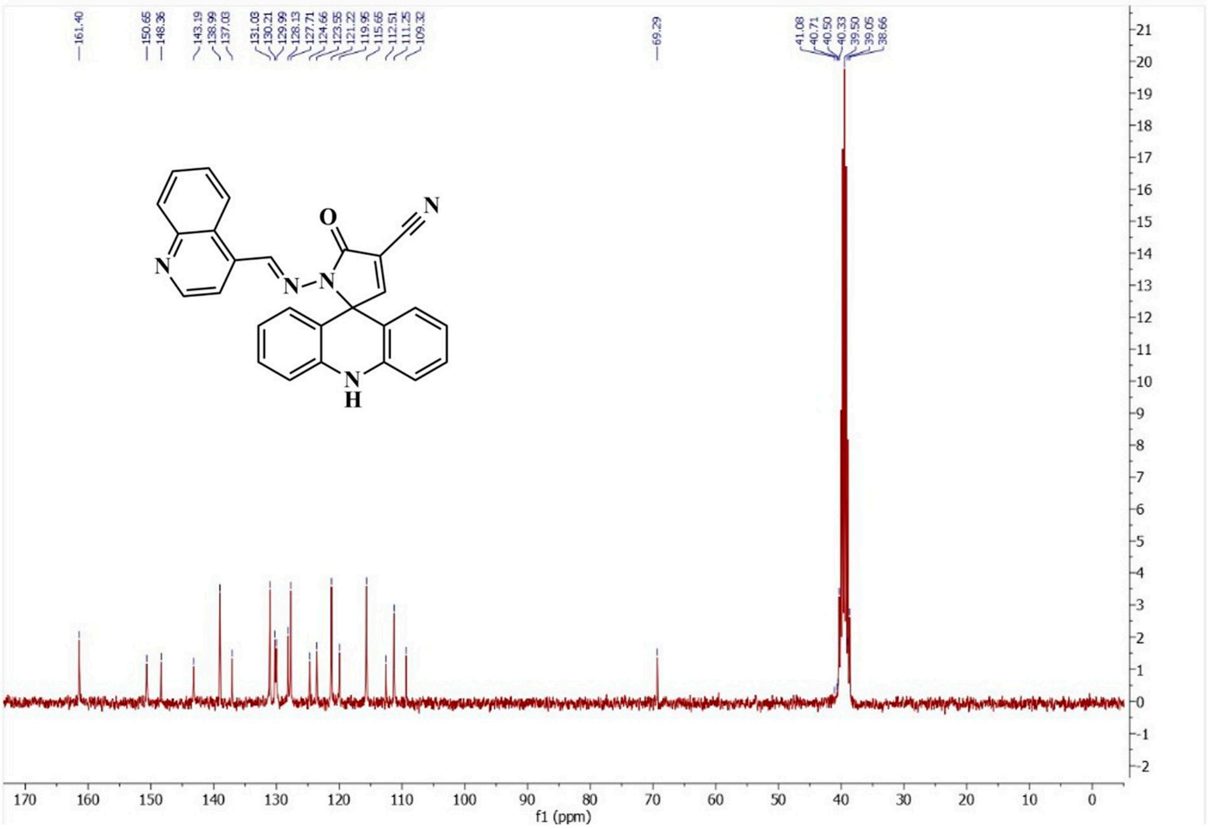

Figure S7. ^{13}C NMR spectrum of AMTAC-21 (100 MHz, BB, DMSO-d_6).

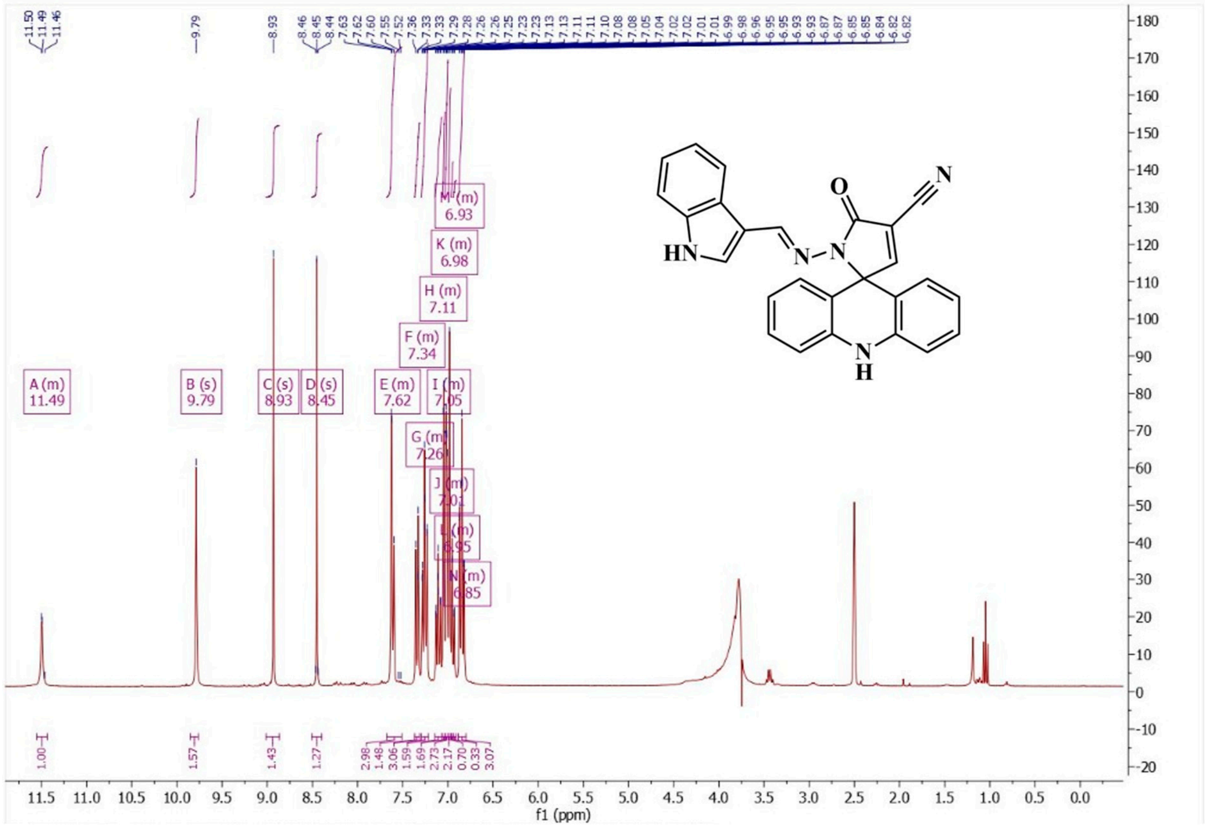

Figure S8. ^{1}H NMR spectrum of AMTAC-22 (400 MHz, DMSO-d_6).

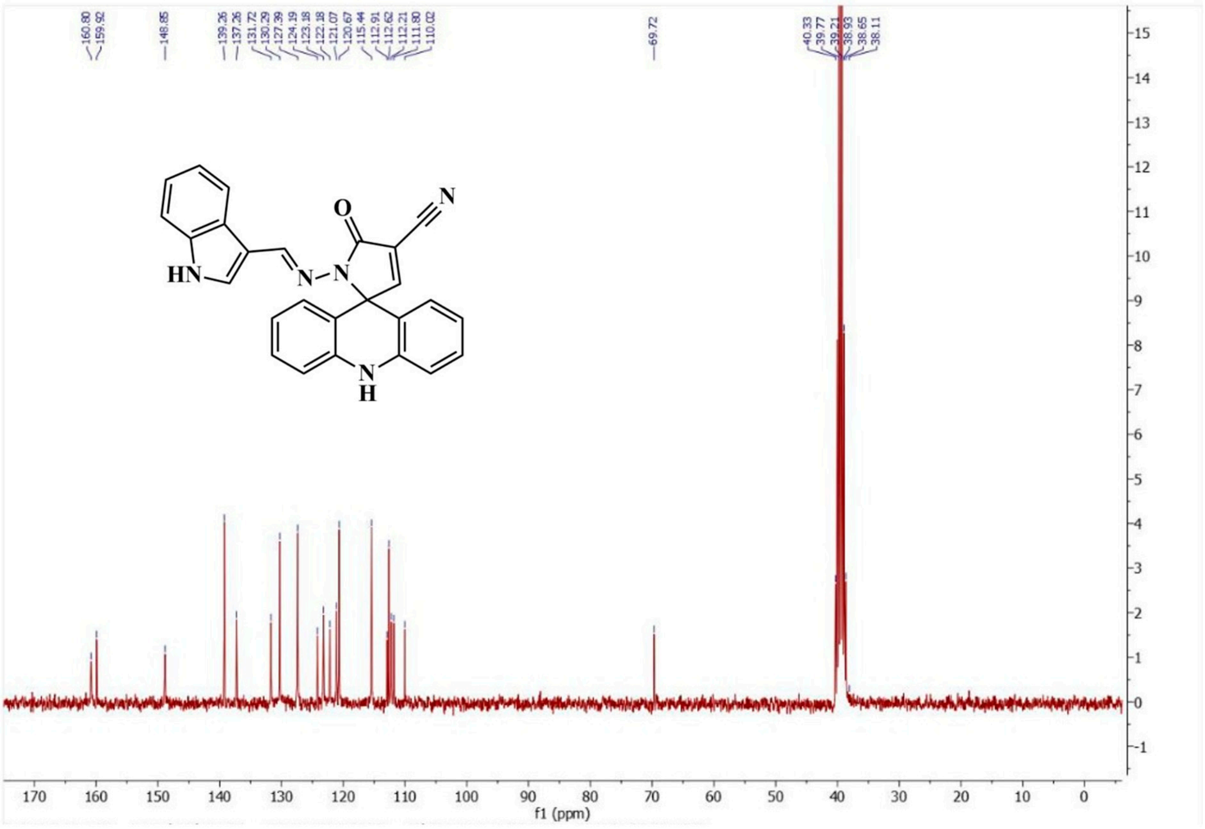

Figure S9. ^{13}C NMR spectrum of AMTAC-22 (100 MHz, BB, DMSO-d_6).

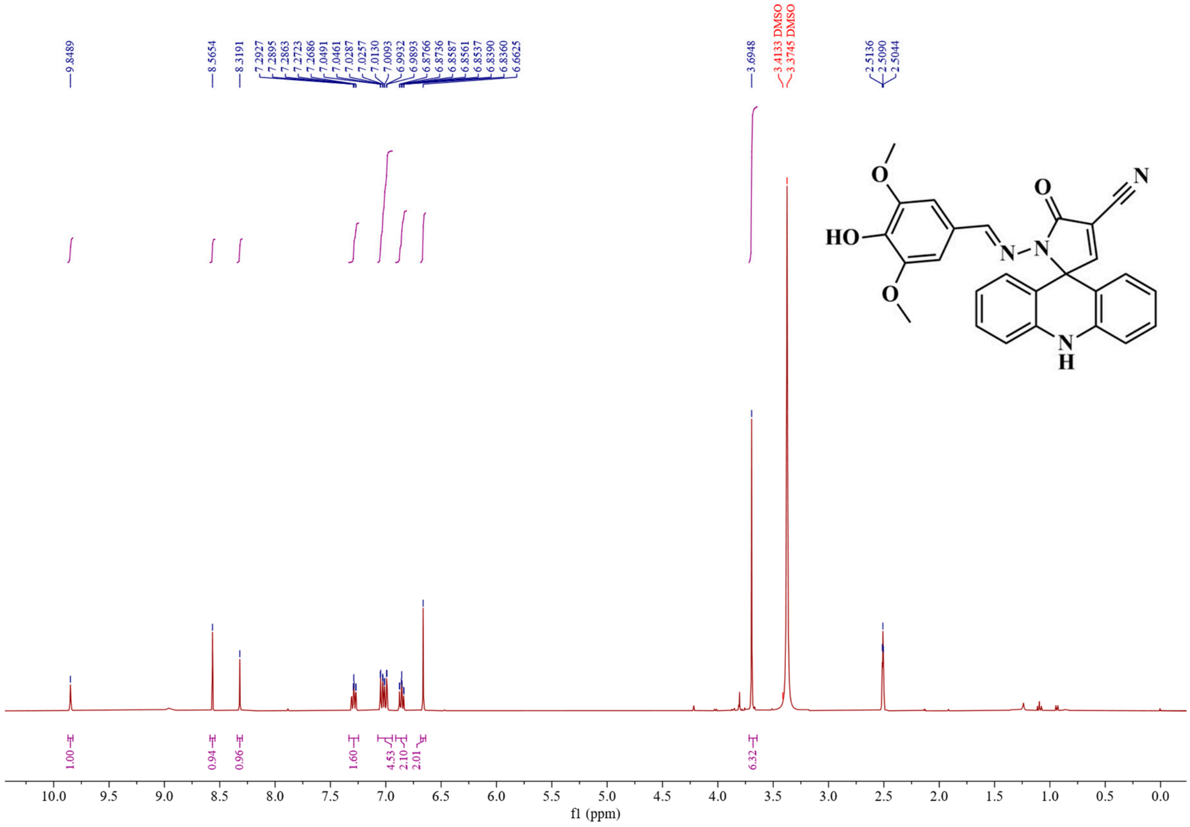

Figure S10. ^{1}H NMR spectrum of AMTAC-24 (400 MHz, DMSO-d_6).

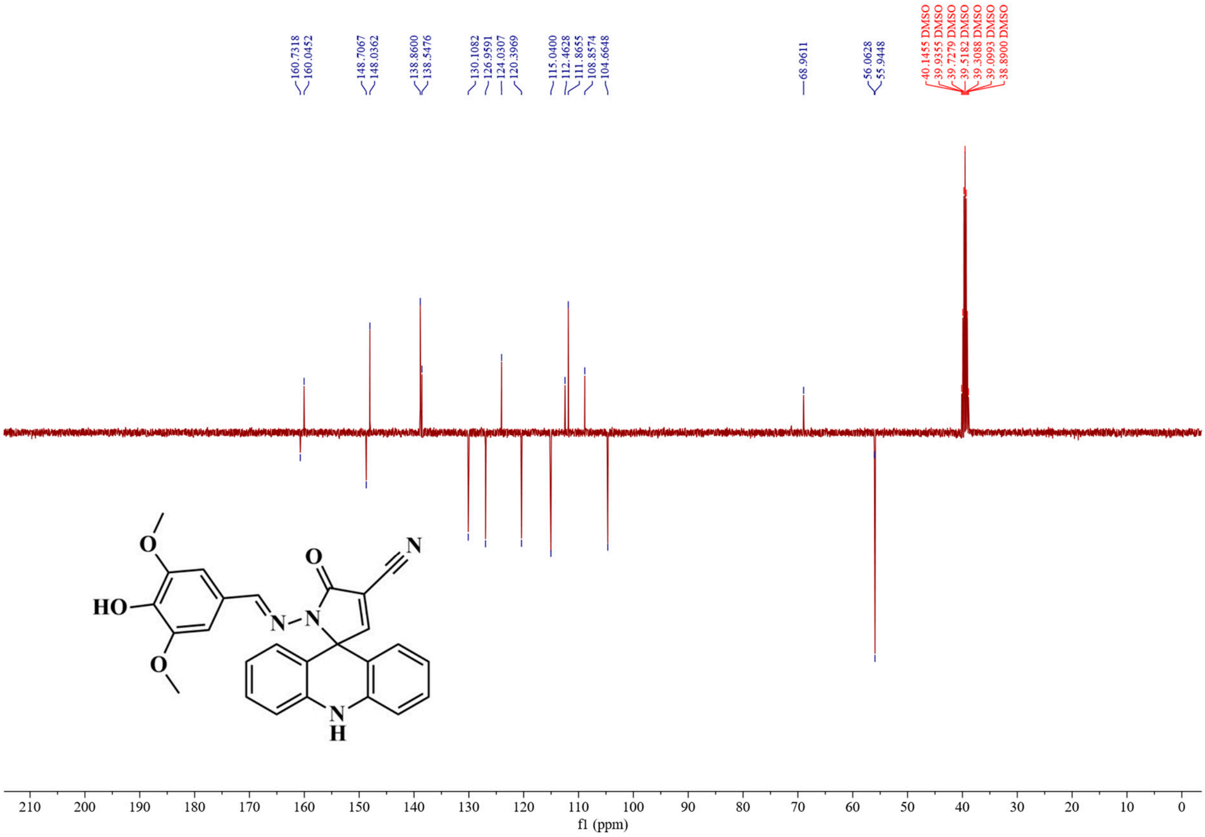

Figure S11. ^{13}C NMR spectrum of AMTAC-24 (100 MHz, APT, DMSO-d_6).

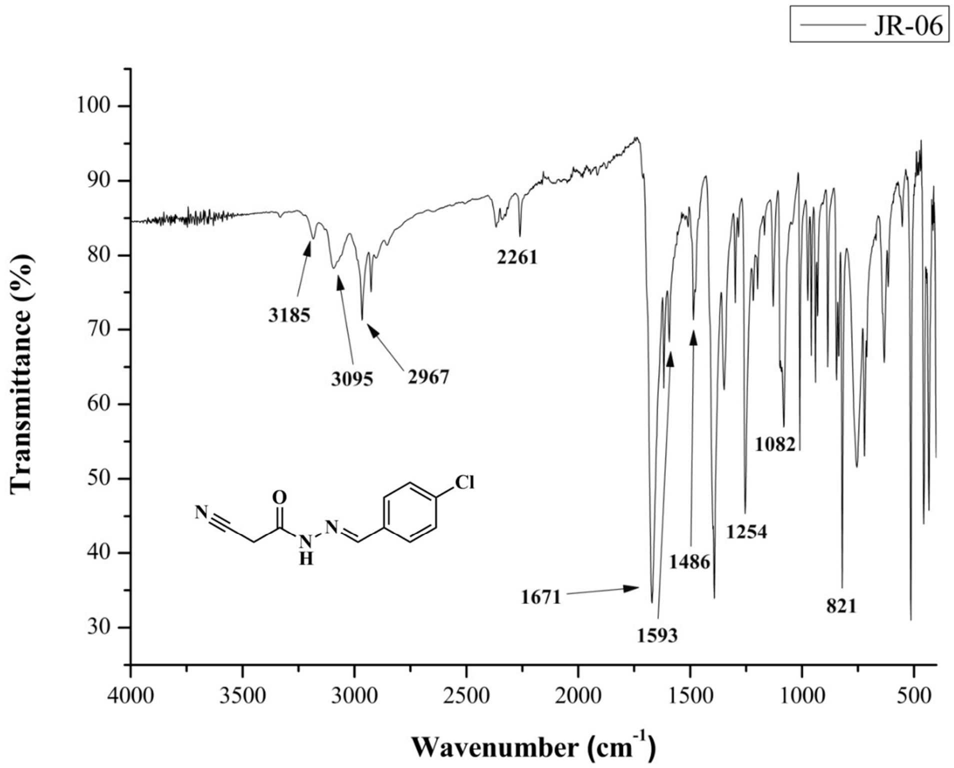

Figure S12. Infrared spectrum of JR-06 (ATR).

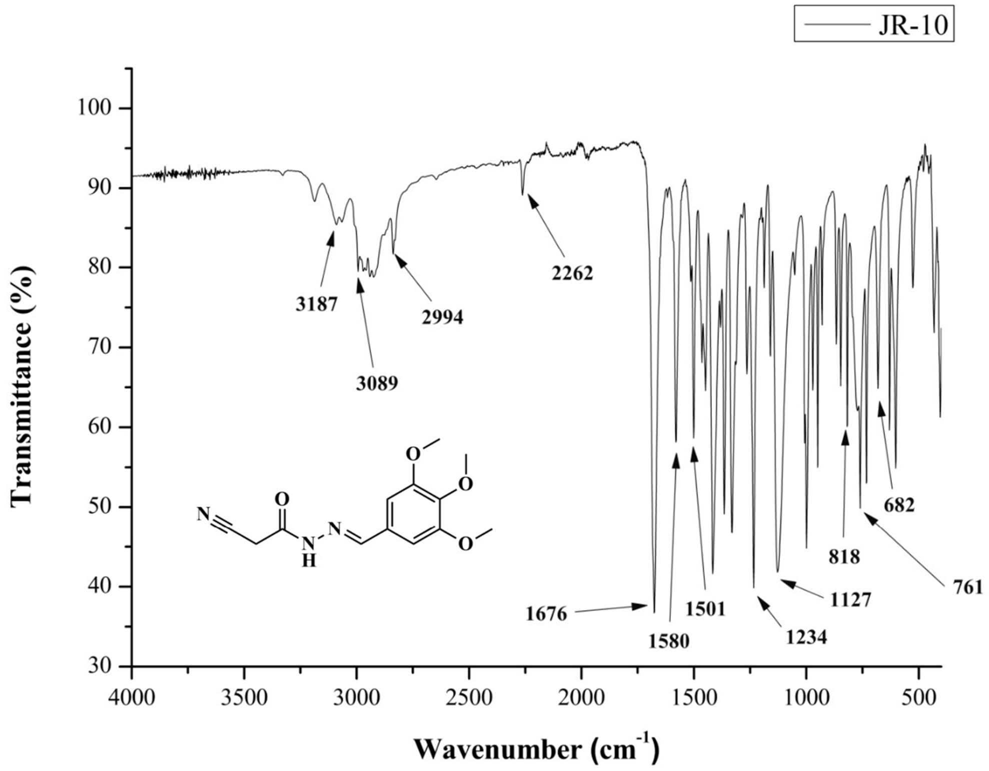

Figure S13. Infrared spectrum of JR-10 (ATR).

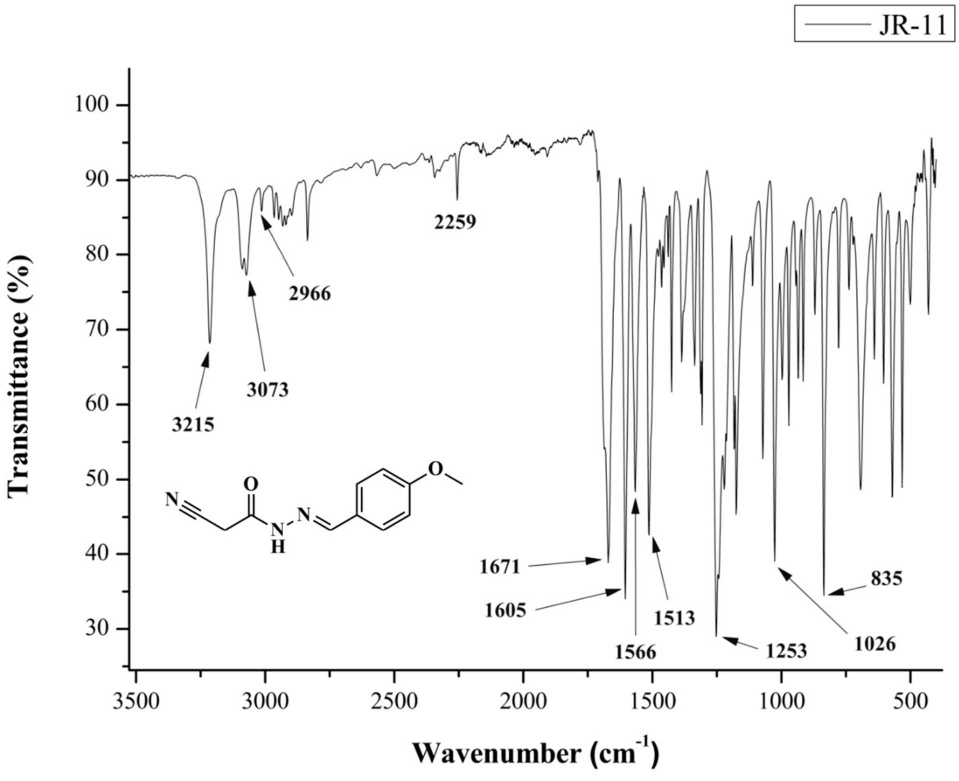

Figure S14. Infrared spectrum of JR-11 (ATR).

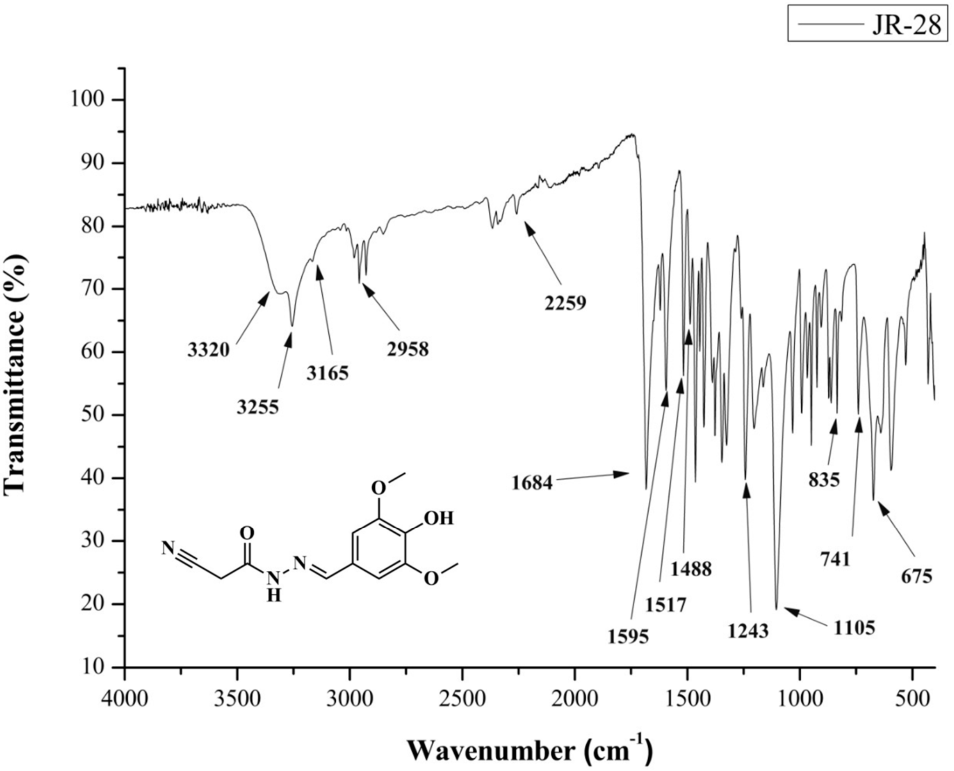

Figure S15. Infrared spectrum of JR-28 (ATR).

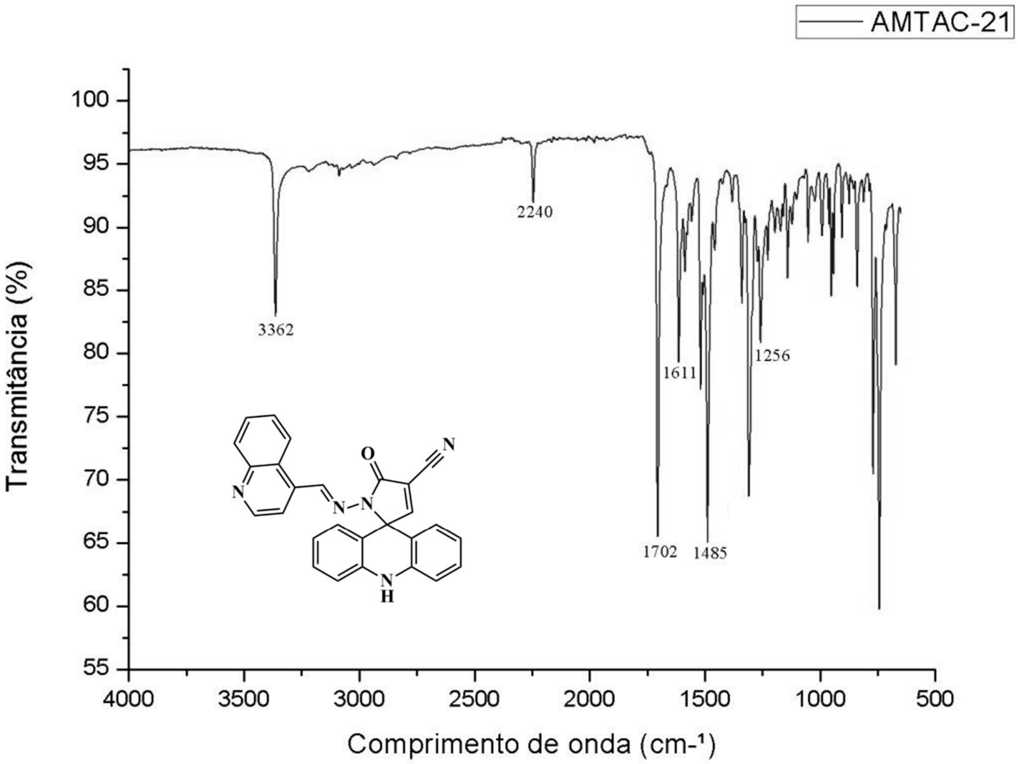

Figure S16. Infrared spectrum of AMTAC-21 (ATR).

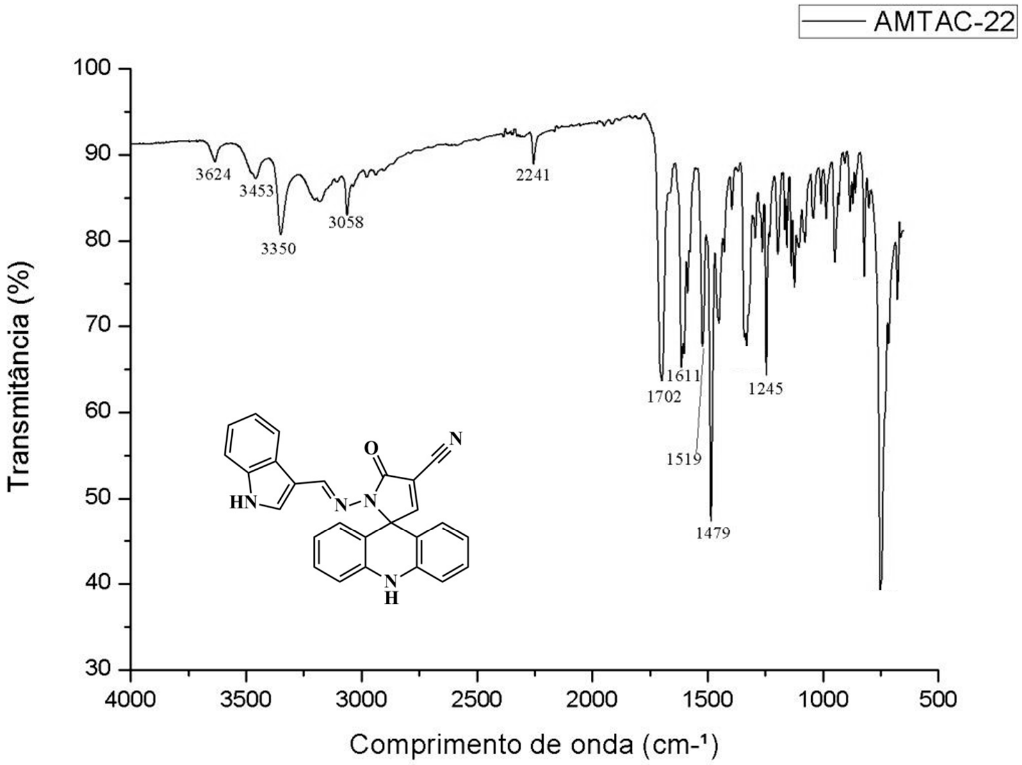

Figure S17. Infrared spectrum of AMTAC-22 (ATR).

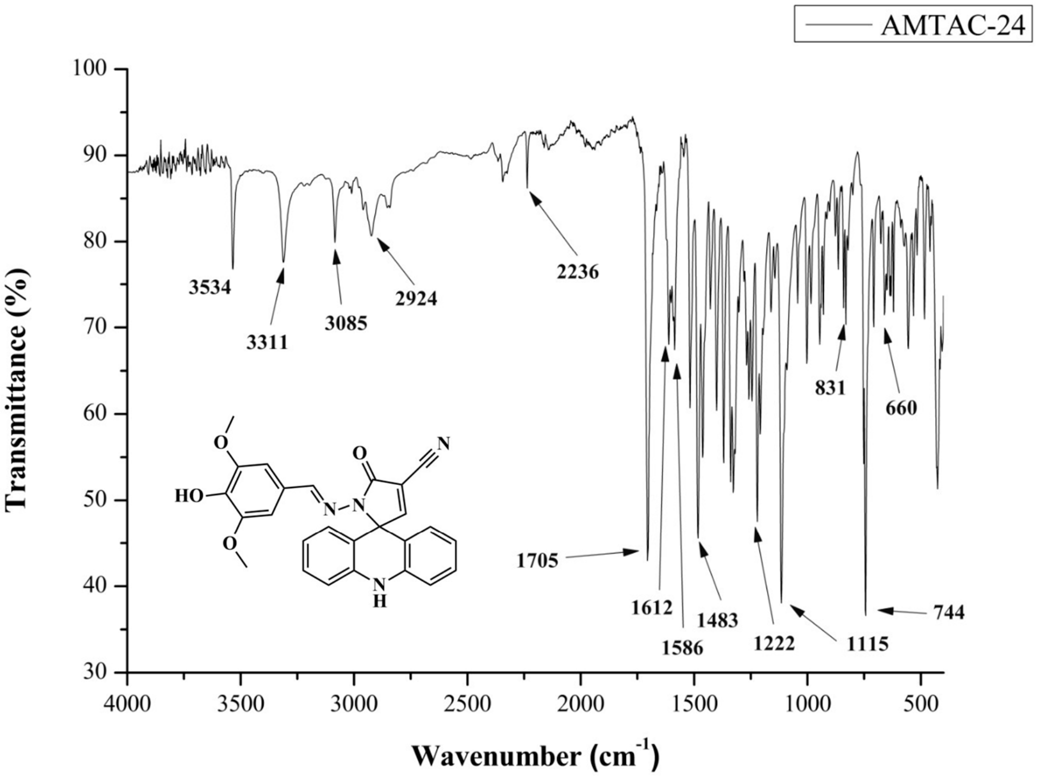

Figure S18. Infrared spectrum of AMTAC-24 (ATR).

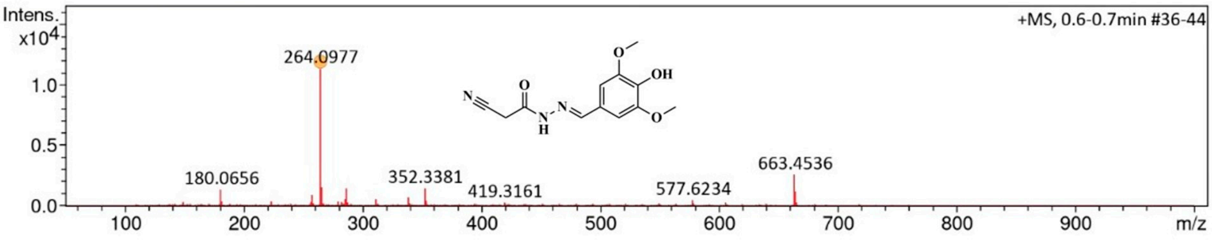

Figure S19. Mass spectrum of JR-28 by HRESIMS.

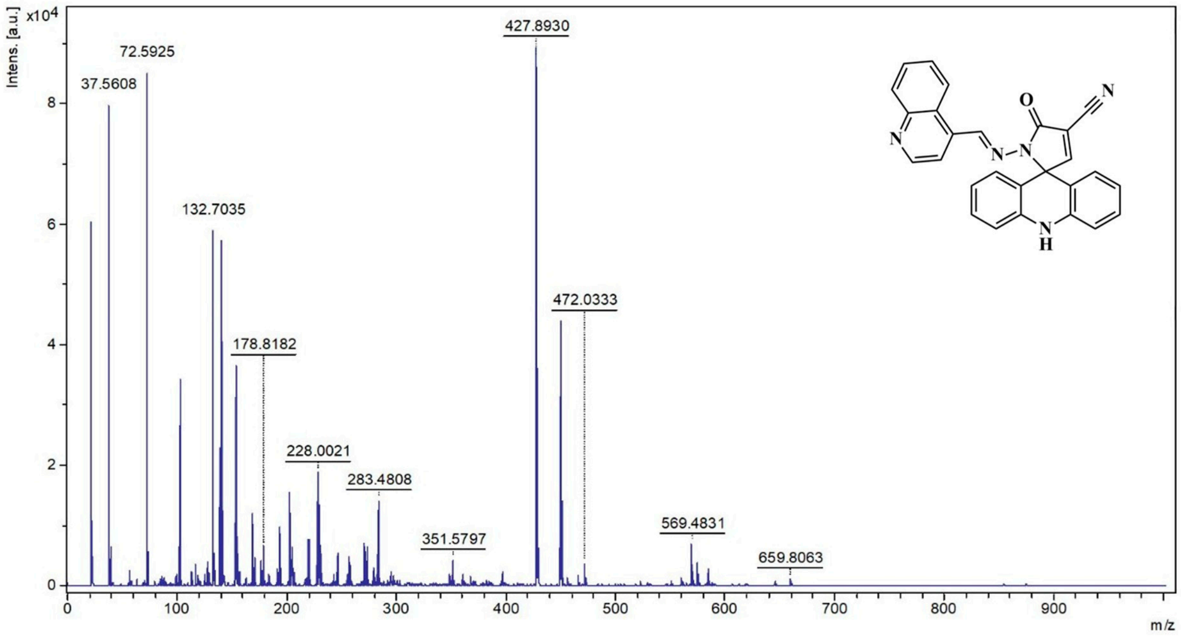

Figure S20. Mass spectrum of AMTAC-21 by MALDI-TOF.

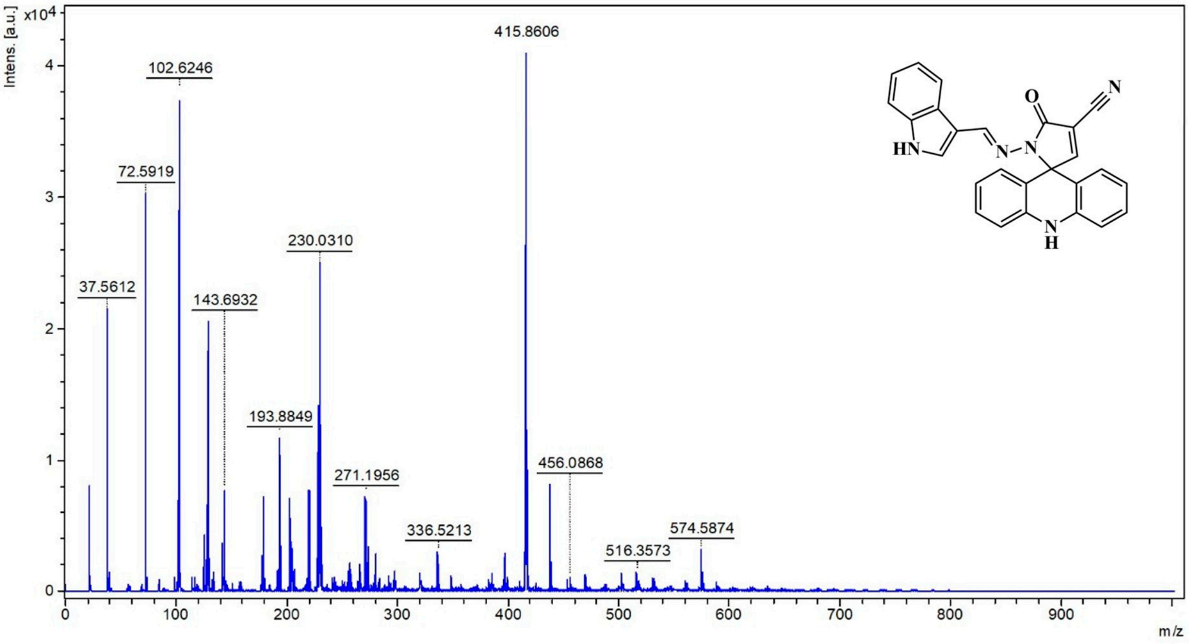

Figure S21. Mass spectrum of AMTAC-22 by MALDI-TOF.

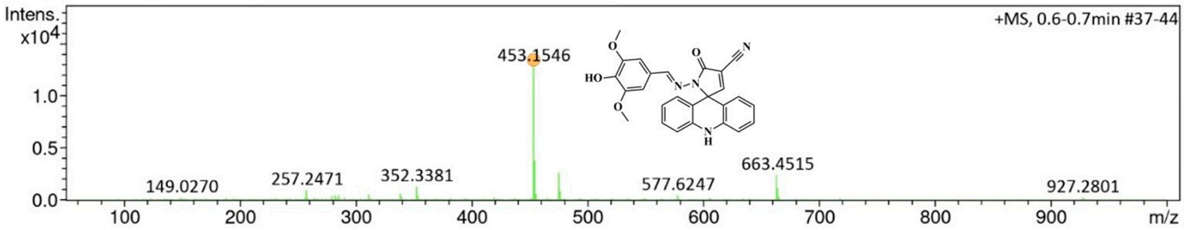

Figure S22. Mass spectrum of AMTAC-24 by HRESIMS.

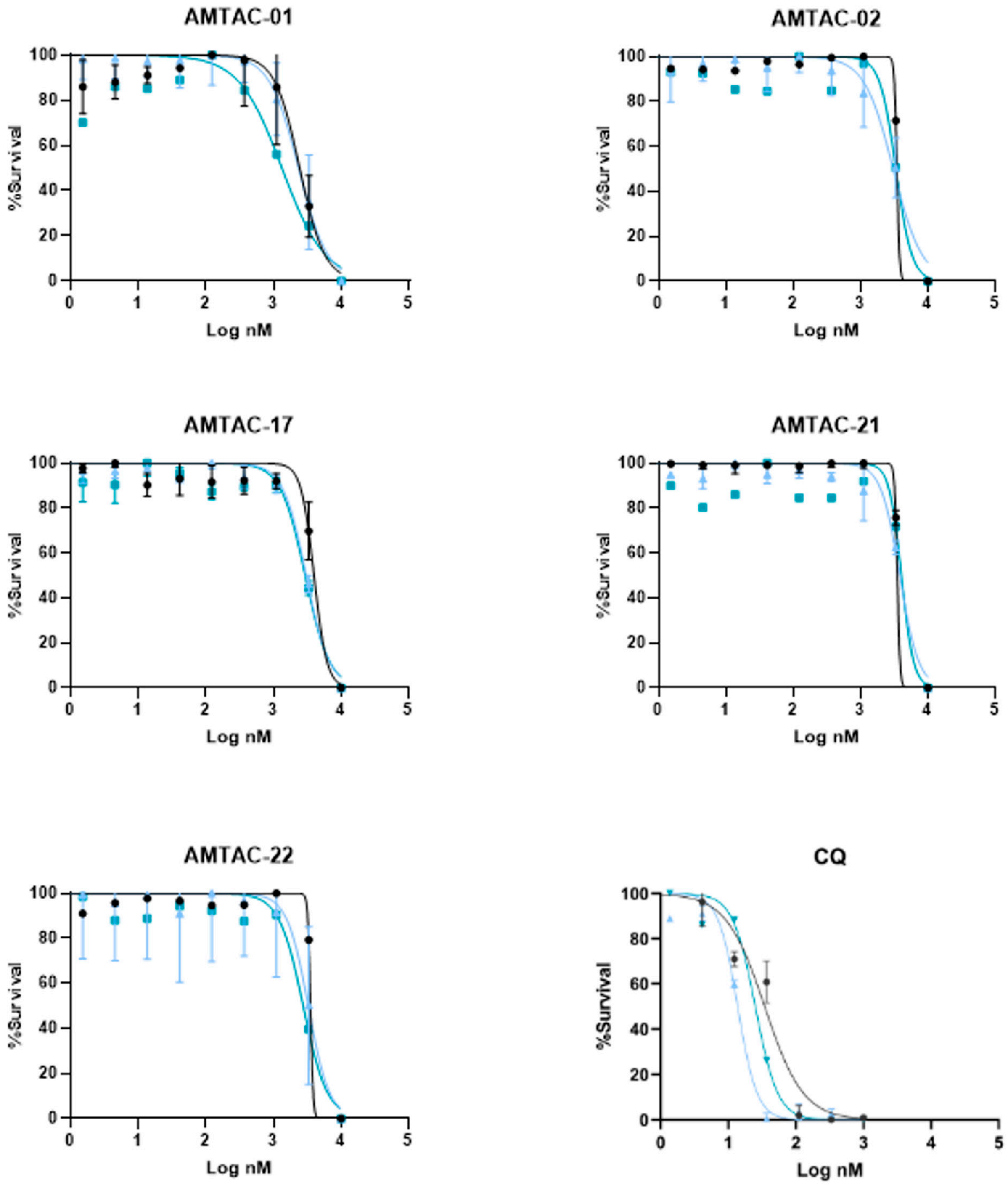

Figure S23. Concentration-response curves of AMTAC compounds and Chloroquine against *Plasmodium falciparum* 3D7-GFP strain.

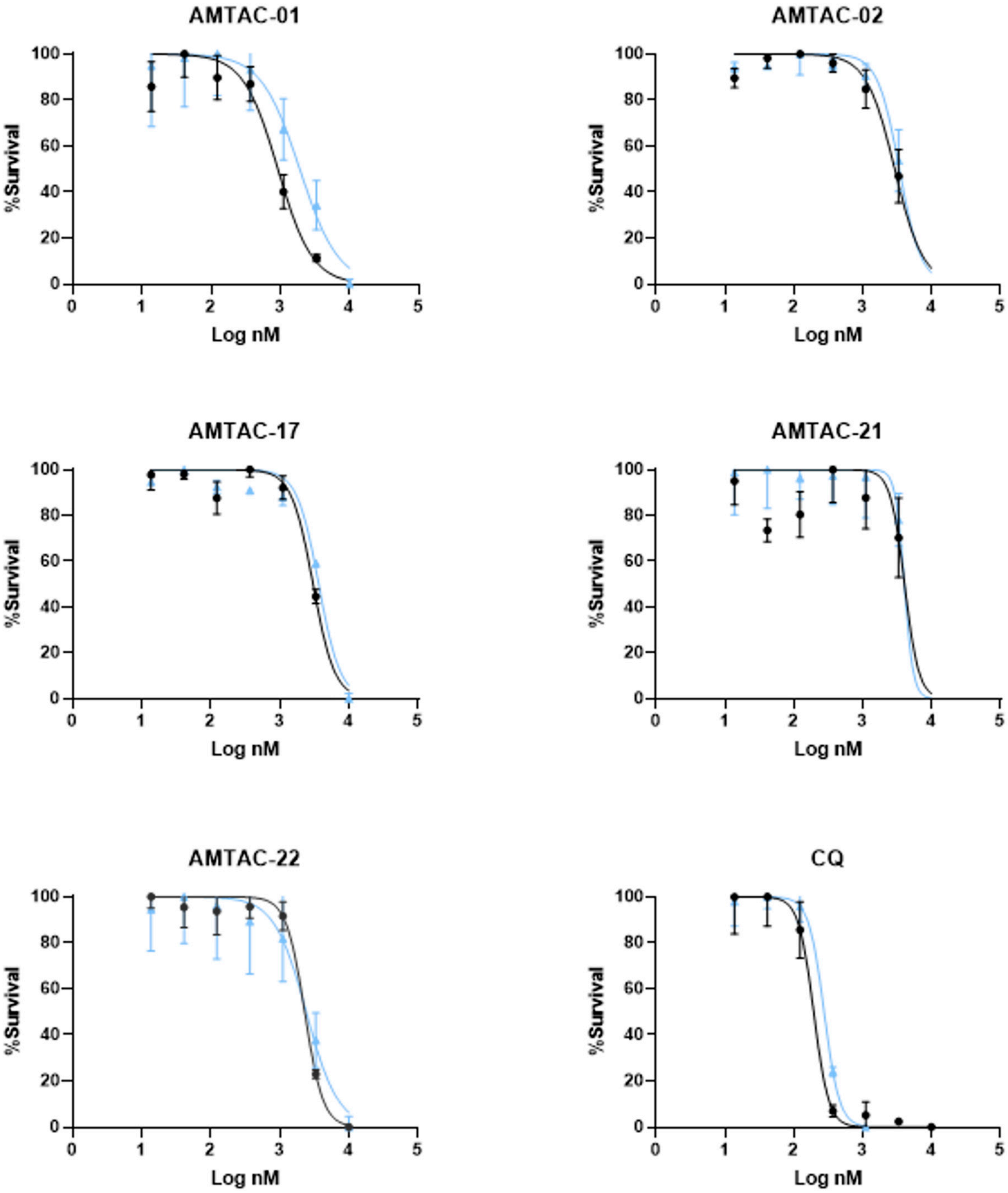

Figure S24. Concentration-response curves of AMTAC compounds and Chloroquine against *Plasmodium falciparum* Dd2 strain.

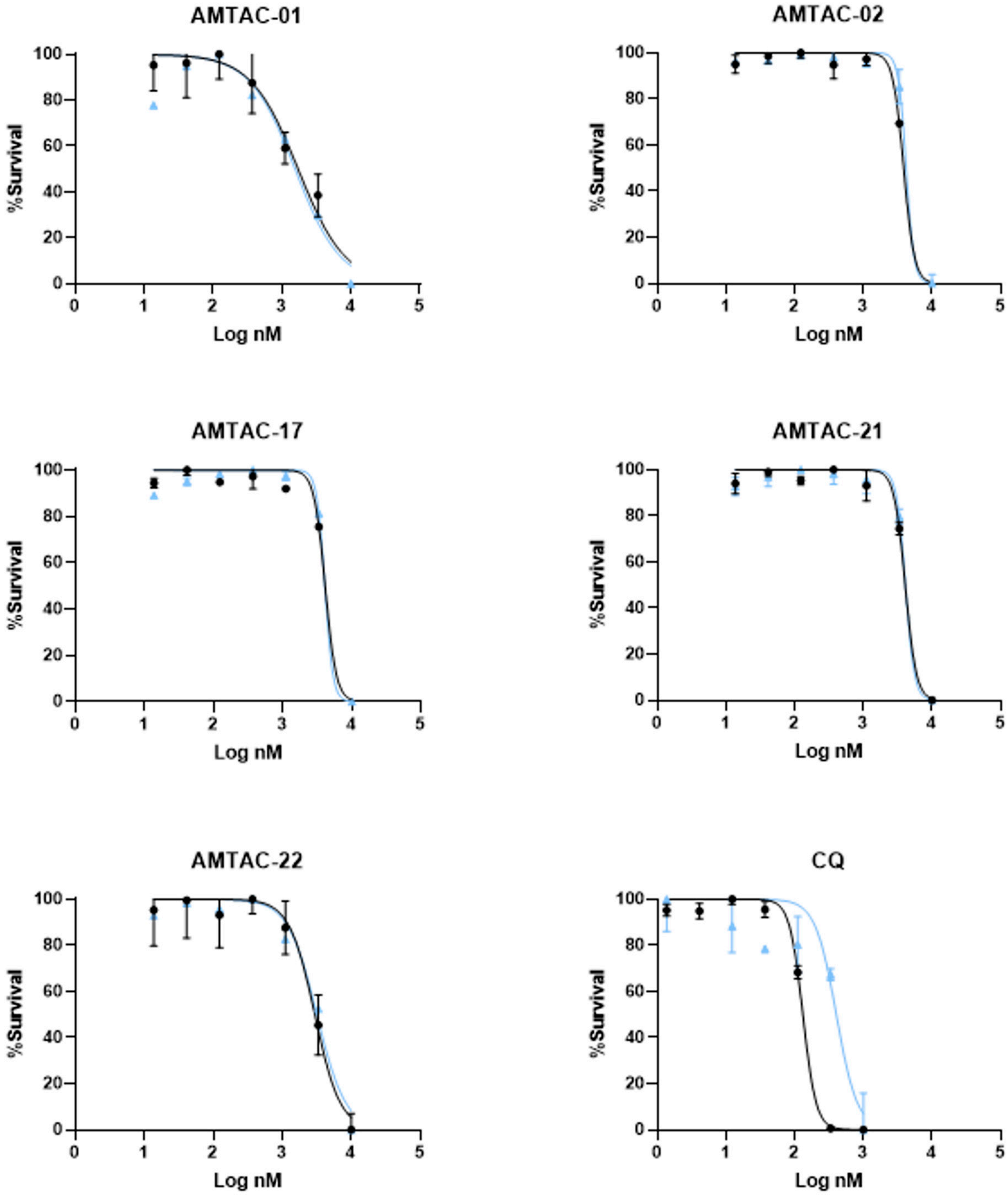

Figure S25. Concentration-response curves of AMTAC compounds and Chloroquine against *Plasmodium falciparum* MRA-1240 strain.
